# Breastfeeding and risk of maternal type 2 diabetes: a prospective cohort study of 280 000 women in China

**DOI:** 10.1136/bmjopen-2025-109377

**Published:** 2026-06-28

**Authors:** Johanna Kaminer, Christiana Kartsonaki, Paul Sherliker, Ling Yang, Andri Iona, Pei Pei, Yiping Chen, Dianjianyi Sun, Xiaoming Yang, Yan Lu, Huaidong Du, Jun Lv, Canqing Yu, Junshi Chen, Liming Li, Zhengming Chen, Fiona Bragg, China Kadoorie Biobank Collaborative Group, Junshi Chen

**Affiliations:** 1 Clinical Trial Service Unit & Epidemiological Studies Unit (CTSU), Nuffield Department of Population Health, University of Oxford, Oxford, UK; 2 Department of Epidemiology & Biostatistics, School of Public Health, Peking University Health Science Center, Beijing, China; 3 Peking University Center for Public Health and Epidemic Preparedness & Response, Peking University, Beijing, China; 4 Key Laboratory of Epidemiology of Major Diseases (Peking University), Ministry of Education, Beijing, China; 5 Suzhou Center for Disease Prevention and Control, Suzhou, Jiangsu, 215004, China, Suzhou, China; 6 China National Center for Food Safety Risk Assessment, Beijing, China; 7 Health Data Research UK Oxford, University of Oxford, Oxford, UK

**Keywords:** China, Diabetes Mellitus, Type 2, EPIDEMIOLOGY

## Abstract

**Abstract:**

**Background:**

Breastfeeding may be associated with lower future risk of maternal type 2 diabetes (T2D). However, existing evidence is inconsistent and derived largely from studies in high-income countries. We assess the association of breastfeeding and breastfeeding duration with incident T2D among Chinese women.

**Research design and methods:**

The prospective China Kadoorie Biobank recruited 512 724 adults from 10 localities across China between 2004 and 2008. During 11.8 years’ follow-up, 12 011 cases of incident T2D were recorded among 283 855 female participants without prior diabetes. Cox regression was used to estimate adjusted HRs for incident T2D associated with ever breastfeeding, mean breastfeeding duration per child and lifetime breastfeeding duration.

**Results:**

Overall, 98.6% of female participants were parous, among whom 97.2% reported ever breastfeeding, with mean lifetime breastfeeding duration and breastfeeding duration per child of 34.8 and 14.9 months, respectively. Among parous female participants, there was no clear association between ever breastfeeding and risk of incident T2D (adjusted HR 1.06 (95% CI 0.94 to 1.20)). A modest log-linear positive association was observed between lifetime breastfeeding duration and incident T2D among parous female participants who ever breastfed (1.01 (1.01 to 1.02) per 6 months longer breastfeeding), but this was attenuated after adjustment for parity (1.00 (0.99 to 1.01)). Mean breastfeeding duration per child was not associated with incident T2D (1.01 (0.99 to 1.02) per 6 months longer breastfeeding).

**Conclusions:**

In this population with almost universal childbearing and breastfeeding, there was no apparent association of ever breastfeeding or of breastfeeding duration with incident T2D.

**STRENGTHS AND LIMITATIONS OF THIS STUDY**

- This study uses data from a prospective cohort of over 280 000 women recruited from both urban and rural areas of China and followed up for an average of 12 years, enabling investigation of the long-term relevance of breastfeeding and breastfeeding duration for future maternal type 2 diabetes (T2D) risk in a large, diverse and relatively understudied population.
- Almost universal childbearing and breastfeeding in the study population, coupled with collection of detailed information on a range of reproductive, sociodemographic and lifestyle-related factors, enabled robust and detailed assessment of the independent relevance of breastfeeding for risk of T2D.
- Similar to previous studies on this topic, information on breastfeeding was self‐reported several years after childbirth, potentially contributing to measurement error, and the very high frequency of breastfeeding in the population precluded assessment of the relevance of breastfeeding initiation in population subgroups.
- Given the age of the study cohort, all incident diabetes cases were assumed to be T2D; however, a small proportion may have been type 1 diabetes.

## Background

 Diabetes is one of the major public health challenges of the twenty-first century. In 2021, an estimated 537 million people, including 259 million women, were living with diabetes worldwide, and the overall number is projected to increase to more than 783 million by 2045.^1^ Reflecting this global trend, a rapid increase in the burden of diabetes has been reported in China in recent decades, with prevalence growing from an estimated 90 million in 2011 to 141 million in 2021.^1^ Several important modifiable risk factors for type 2 diabetes (T2D), which accounts for approximately 90% of all diabetes cases, are well established, most notably excess adiposity.^2^ However, a sizeable proportion of T2D risk, and of variation in T2D risk within and between populations, remains incompletely understood.^3^

Breastfeeding has been suggested to influence future T2D risk among mothers. More specifically, studies conducted predominantly in high-income countries have observed up to 32% lower risk of future T2D among women who have breastfed,^4 5^ with longer breastfeeding duration associated with lower T2D risk.^6^ Based on such studies, maternal T2D prevention is listed among the health benefits of breastfeeding in national and international guidance.^7^,^9^ However, much of the existing evidence is based on relatively small studies, with inconsistent findings.^610^,^13^ Moreover, the extent to which the reported modest benefits of breastfeeding for maternal T2D risk reduction are independent of important potential confounding factors, such as socioeconomic position, adiposity and other female reproductive factors, remains unclear.

Female reproductive patterns in the Chinese population differ from those in Western populations, including less childlessness and almost universal breastfeeding among parous Chinese women, especially among older generations.^14^ Moreover, correlates of breastfeeding and breastfeeding duration, including associations with socioeconomic position and with measures of adiposity, appear to differ.^14^ Studying the association of breastfeeding with maternal T2D risk in Chinese populations may therefore provide novel insights into this relationship. However, there is currently limited evidence about the long-term relevance of breastfeeding for risk of maternal T2D in Chinese populations, with existing studies frequently limited by small sample size, cross-sectional design or restriction to urban cohorts.^15^,^17^

To address these evidence gaps, we assess the associations of breastfeeding and breastfeeding duration with risk of maternal incident T2D within the nationwide prospective China Kadoorie Biobank (CKB) study.

## Methods

### China Kadoorie Biobank study design and population

The CKB study design and population have been described previously.^18 19^ In brief, between 2004 and 2008, 210 205 men and 302 519 women aged 30–79 years were recruited from 10 diverse areas—5 urban and 5 rural—across China (online supplemental figure S1).

### Data collection

During baseline assessments at study clinics, trained health workers administered laptop-based questionnaires collecting data on demographic and socioeconomic characteristics, lifestyle factors (including smoking, alcohol consumption, diet and physical activity) and personal and family medical history. Among women, data were additionally collected on reproductive factors, including age at menarche, number of pregnancies and live births and, among parous women, age at the time of these births and breastfeeding duration, as well as menopausal status and, among post-menopausal women, age at menopause. Physical measurements including blood pressure and anthropometric measurements were taken using calibrated instruments with standard methods. Weight was measured using a body composition analyser (TANITA-TBF-300GS; Tanita Corporation, Tokyo, Japan), and standing height was measured with a stadiometer to the nearest 0.1 cm. Body mass index (BMI) was calculated as weight in kilogrammes divided by the square of standing height in metres (kg/m^2^). Waist circumference was measured to the nearest 0.1 cm using a non-stretchable tape. Non-fasting blood samples were collected into ethylenediaminetetraacetic acid vacutainers for long-term storage and immediate onsite random plasma glucose testing using the SureStep Plus system (LifeScan, Milpitas, California, USA).

### Assessment of breastfeeding duration

Information collected on reproductive history among parous women included number of live births and breastfeeding duration for each child. Lifetime breastfeeding duration was calculated as the sum of the breastfeeding duration for each child. Mean breastfeeding duration per child was defined as lifetime breastfeeding duration divided by number of live births.

### Follow-up for incident type 2 diabetes

Participants are followed for cause-specific morbidity and mortality through linkage with established regional disease registries (including for diabetes) and with death registries, and with the national health insurance system via participants’ unique national identification numbers. Data linkage with the national health insurance system occurs every 6 months to retrieve information on International Statistical Classification of Diseases and Related Health Problems, 10th Revision (ICD-10)-coded diagnoses resulting in or during hospitalisation. The vital status of participants is monitored through death registries and checked annually against local residential and health insurance records and by active confirmation. Deaths are ICD-10 coded by trained staff blinded to baseline information. Cases of incident T2D were identified through the disease surveillance system for diabetes and through diabetes diagnoses (ICD-10 codes E10–E14) recorded in the health insurance databases or as underlying or contributing to death on death certificates. By 1 January 2019, <1% (n=4028) of participants were lost to follow-up.

### Statistical analyses

The present study excluded men (n=210 205), women with missing BMI (n=1) or reproductive history (n=47) data and women with self-reported previously diagnosed diabetes or screen-detected diabetes (no self-reported diabetes and plasma glucose ≥7.0 mmol/L with fasting time ≥8 hours, plasma glucose ≥11.1 mmol/L with fasting time <8 hours, or fasting plasma glucose ≥7.0 mmol/L^20^) at recruitment (n=18 616). Overall, 283 855 women remained for inclusion in the main analyses.

Prevalence and mean values of baseline characteristics were calculated across categories of breastfeeding initiation and duration, standardised by 5-year age groups and study area. In order to assess the shape of the association of breastfeeding duration with risk of incident T2D, lifetime breastfeeding duration (1–12/13–24/25–36/37–48/>48 months) and breastfeeding duration per child (<7/7–12/13–18/19–24/>24 months, respectively) were each classified into five groups. Cox proportional hazards models with time since entry into the study as the underlying timescale estimated HRs and 95% CIs for incident T2D associated with ever breastfeeding, lifetime breastfeeding duration and breastfeeding duration per child. Models were stratified by age-at-risk (5-year age groups) and study area (10 areas) and adjusted for education (no formal education/primary school/middle school/high school/college/university); household income (<2500/2500–4999/5000–9999/10 000–19 999/20 000–34 999/≥35 000 yuan); smoking (never smoker/occasional smoker/ex-regular smoker/current smoker); alcohol drinking (never regular drinker/ex-regular drinker/occasional or seasonal drinker/monthly drinker/reduced intake/weekly drinker); physical activity (<10.9/10.9–17.1/17.2–28.6/≥28.7 metabolic equivalent of task hours per day); consumption (daily/4–6 days per week/1–3 days per week/monthly/never or rarely) of fresh fruit, meat, soybean products, rice and wheat; and family history of diabetes. This approach allows for a different baseline hazard in each stratum, such that the proportional hazards assumption was made only within strata of age group and study area. Subsequent analyses additionally adjusted for parity (1/2/3/4/>4 children), BMI (<18.5/18.5–24.9/25.0–29.9/≥30 kg/m^2^) and waist circumference (<72.0/72.0–77.9/78.0–84.5/≥84.6 cm). Separate Cox regression analyses examined the associations with incident T2D of other reproductive factors, specifically age at menarche, number of live births, age at first birth, age at menopause and reproductive years. In analyses involving more than two exposure categories, CIs were estimated using the floating absolute risks method, deriving CIs from the variance of the log risk in each category and allowing comparisons between any two categories rather than just with the reference group.^21^

Adjusted HRs for incident T2D associated with breastfeeding duration were examined separately for female participants in urban and rural areas and in strata of birth cohort, BMI and other reproductive factors, including number of live births, age at first birth and menopause status. Sensitivity analyses examined the relevance of breastfeeding duration for risk of incident T2D after additional adjustment for birth cohort (<1950/1950–1959/≥1960) and for other reproductive factors.

Analyses were undertaken using R V.4.3.0.

### Patient and public involvement

Patients were not involved in the present study. The results of the main study were presented to study participants on the website of the CKB study (http://www.ckbiobank.org/site/) and by newsletters annually.

## Results

Among 283 855 female participants included in the present analyses, 98.6% (n=280 003) were parous, with a mean (SD) of 2.2 (1.3) live births. Compared with parous female participants, nulliparous female participants had a similar mean (SD) age at recruitment (49.6 (11.7) vs 51.0 (11.7) years, respectively), but were more likely to live in urban areas, to be more highly educated but have a lower average household income and to report a history of miscarriage or stillbirth (table 1). Among parous female participants, 2.8% (n=7724) reported never breastfeeding. These female participants were, on average, younger at the time of recruitment than parous female participants who ever breastfed (46.8 (SD 9.4) vs 51.1 (10.4) years, respectively) and were more likely to live in urban areas, to be more highly educated with a higher average household income and to have an older average age at first birth.

**Table 1:** Baseline characteristics of study participants by lifetime breastfeeding duration

Characteristic*	Nulliparous	Parous female participants						
		Never breastfed	Lifetime breastfeeding duration, months					
			1–12	13–24	25–36	37–48	>48	Total
No. of participants	3852	7724	71 218	69 359	42 167	30 866	58 669	272 279
Age and socioeconomic factors								
Mean age (SD), years	51.0 (11.7)	46.8 (9.4)	43.9 (7.2)	47.7 (8.6)	53.6 (10.0)	57.2 (11.0)	62.7 (10.1)	51.1 (10.4)
Living in rural area, %	37.7	28.3	28.8	49.9	69.5	78.5	89.0	57.4
6+ years of education, %	46.8	55.6	54.9	46.6	38.6	31.5	26.3	43.5
Annual household income ≥20 000 yuan, %	34.1	45.3	46.6	42.6	38.5	36.5	30.2	40.6
Lifestyle factors								
Ever smoker, %	5.8	4.2	4.1	4.5	4.9	5.6	7.5	4.9
Ever regular alcohol drinker, %	3.6	2.7	2.7	2.7	3.0	3.4	4.3	3.0
Mean physical activity (SD), MET-h/d	19.8 (11.7)	18.9 (12.0)	20.2 (13.2)	20.8 (13.2)	20.8 (12.3)	21.0 (12.5)	19.6 (12.0)	20.8 (12.8)
Regular consumption†, %								
Fresh fruit	31.7	38.4	37.5	32.4	29.1	27.8	26.3	31.8
Meat	39.9	44.1	46.3	44.6	43.3	43.0	40.9	44.0
Soybean products	9.6	11.1	11.8	9.6	8.5	7.1	8.7	9.1
Rice	71.6	73.2	72.9	72.7	72.3	72.1	72.5	72.4
Wheat	46.0	48.2	47.2	45.1	43.6	44.4	40.2	44.7
Anthropometry, blood pressure and plasma glucose, mean (SD)								
BMI, kg/m^2^	23.6 (3.7)	23.5 (3.4)	23.5 (3.2)	23.8 (3.4)	23.9 (3.5)	24.0 (3.5)	23.5 (3.7)	23.7 (3.4)
WC, cm	78 (10)	78 (9)	78 (9)	79 (9)	79 (10)	80 (10)	78 (10)	79 (9)
RPG, mmol/L	5.8 (1.1)	5.7 (1.0)	5.7 (1.0)	5.7 (1.1)	5.8 (1.1)	5.8 (1.2)	5.6 (1.2)	5.7 (1.1)
SBP, mm Hg	130 (23)	128 (20)	128 (18)	129 (20)	130 (21)	131 (22)	129 (24)	129 (22)
Reproductive history								
Mean age at menarche (SD), years	15.5 (2.6)	14.9 (1.9)	14.9 (1.8)	15.3 (1.9)	15.7 (2.0)	16.0 (2.0)	16.1 (2.0)	15.5 (2.0)
Mean number of live births (SD)	NA	1.8 (0.9)	1.4 (0.4)	1.8 (0.6)	2.3 (0.7)	2.6 (0.9)	3.1 (1.4)	2.2 (1.3)
Mean age at first birth (SD), years	NA	25.1 (3.8)	24.6 (3.1)	23.9 (3.0)	23.0 (2.8)	22.4 (2.7)	21.5 (2.6)	23.4 (3.1)
History of miscarriage, %	39.1	10.6	8.3	8.7	8.9	9.3	10.2	8.9
History of induced abortion, %	54.0	52.7	57.9	53.7	51.3	48.1	46.6	52.4
History of stillbirth, %	19.4	6.3	5.5	5.6	5.7	6.5	6.4	5.6
Mean lifetime breastfeeding duration (SD), months	NA	NA	9.4 (3.0)	20.6 (3.9)	32.4 (3.9)	44.6 (3.7)	67.9 (28.3)	34.8 (29.4)
Mean breastfeeding duration per child (SD), months	NA	NA	8.1 (3.2)	13.2 (4.3)	16.0 (5.2)	19.5 (6.1)	24.7 (8.4)	14.9 (7.3)
Mean age at menopause‡ (SD), years	45.2 (7.1)	46.8 (4.8)	46.8 (4.5)	47.6 (4.2)	48.3 (4.2)	48.4 (4.3)	48.4 (4.4)	48.2 (4.4)
Medical history, %								
Cancer	0.8	0.6	0.7	0.5	0.5	0.4	0.4	0.5
Stroke/TIA	1.1	1.0	1.0	1.1	1.1	1.3	1.3	1.1
Coronary heart disease	2.6	4.0	3.0	3.0	2.8	2.9	2.9	2.8
Chronic kidney disease	1.6	2.1	1.7	1.6	1.5	1.4	1.7	1.6
Poor self-rated health	13.3	15.7	11.3	10.2	10.6	11.4	11.6	10.5
Family history of diabetes	7.8	8.4	7.4	6.5	5.4	5.1	4.4	6.3

* Standardised, where appropriate to age, and study area structure of the study population.

† Defined as ≥4 days per week.

‡ Among 144 069 post-menopausal female participants.

BMI, body mass index; MET-h/d, metabolic equivalent of task hours per day; RPG, random plasma glucose; SBP, systolic blood pressure; TIA, transient ischaemic attack; WC, waist circumference.

Among 272 279 parous female participants who reported ever breastfeeding, the mean (SD) lifetime breastfeeding duration and breastfeeding duration per child were 34.8 (29.4) months and 14.9 (7.3) months, respectively (table 1). Female participants with longer lifetime breastfeeding duration were typically older at recruitment and were more likely to live in rural areas and to be less highly educated with a lower average household income. They more frequently ever smoked tobacco or drank alcohol (although both were uncommon among female participants in the study) and reported less frequent consumption of fresh fruit, meat and wheat. The average age at menarche was typically older among female participants with longer lifetime breastfeeding duration, who, on average, also reported younger age at first birth, older age at menopause and a higher number of live births. Moreover, female participants with longer breastfeeding duration were less likely to have a family history of diabetes. The associations of mean breastfeeding duration per child were similar to those of lifetime breastfeeding duration (online supplemental table S1).

**Figure 1:**
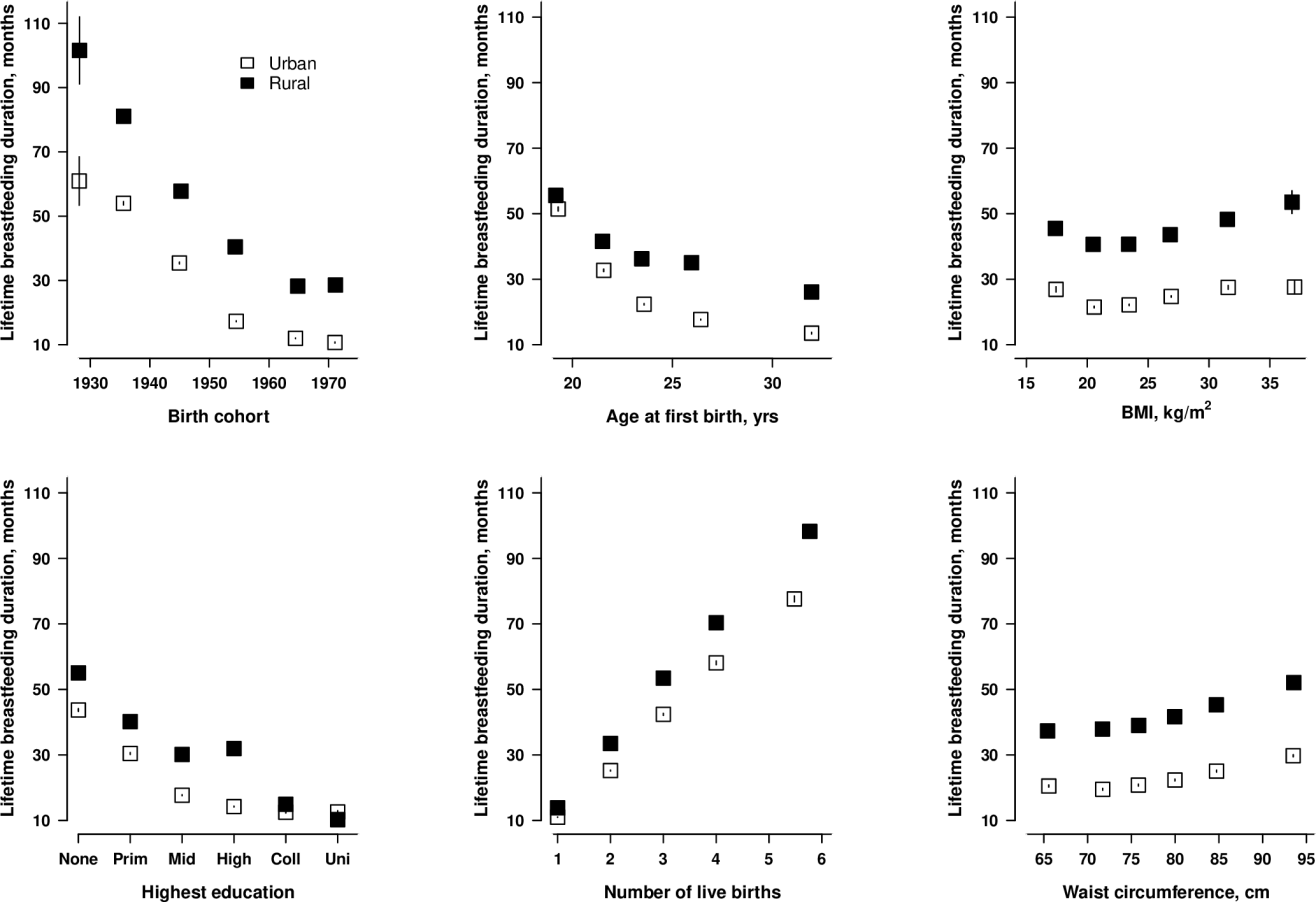
Lifetime breastfeeding duration by birth cohort, education, age at first birth, number of live births and adiposity. Squares represent mean breastfeeding duration by group. Vertical lines represent 95% CIs of the mean. BMI, body mass index.

Overall, lifetime breastfeeding duration was higher among female participants living in rural than in urban areas (figure 1). Among female participants born prior to 1955, there was a clear cohort effect in both urban and rural areas, with participants born earlier typically breastfeeding for longer. Participants who were older at the time of their first birth and who had higher education tended to breastfeed for a shorter period during their lifetimes. There was a J-shaped relationship of BMI with lifetime breastfeeding duration. The shortest average lifetime breastfeeding durations—21.5 months and 40.6 months in urban and rural areas, respectively—were associated with BMI levels between 18.5 and 21.9 kg/m^2^, increasing to 26.9 months and 45.5 months, respectively, at BMI <18.5 kg/m^2^ and to 27.6 months and 53.5 months, respectively, at BMI ≥35 kg/m^2^. A similar shape of association was observed between waist circumference and lifetime breastfeeding duration.

**Figure 2:**
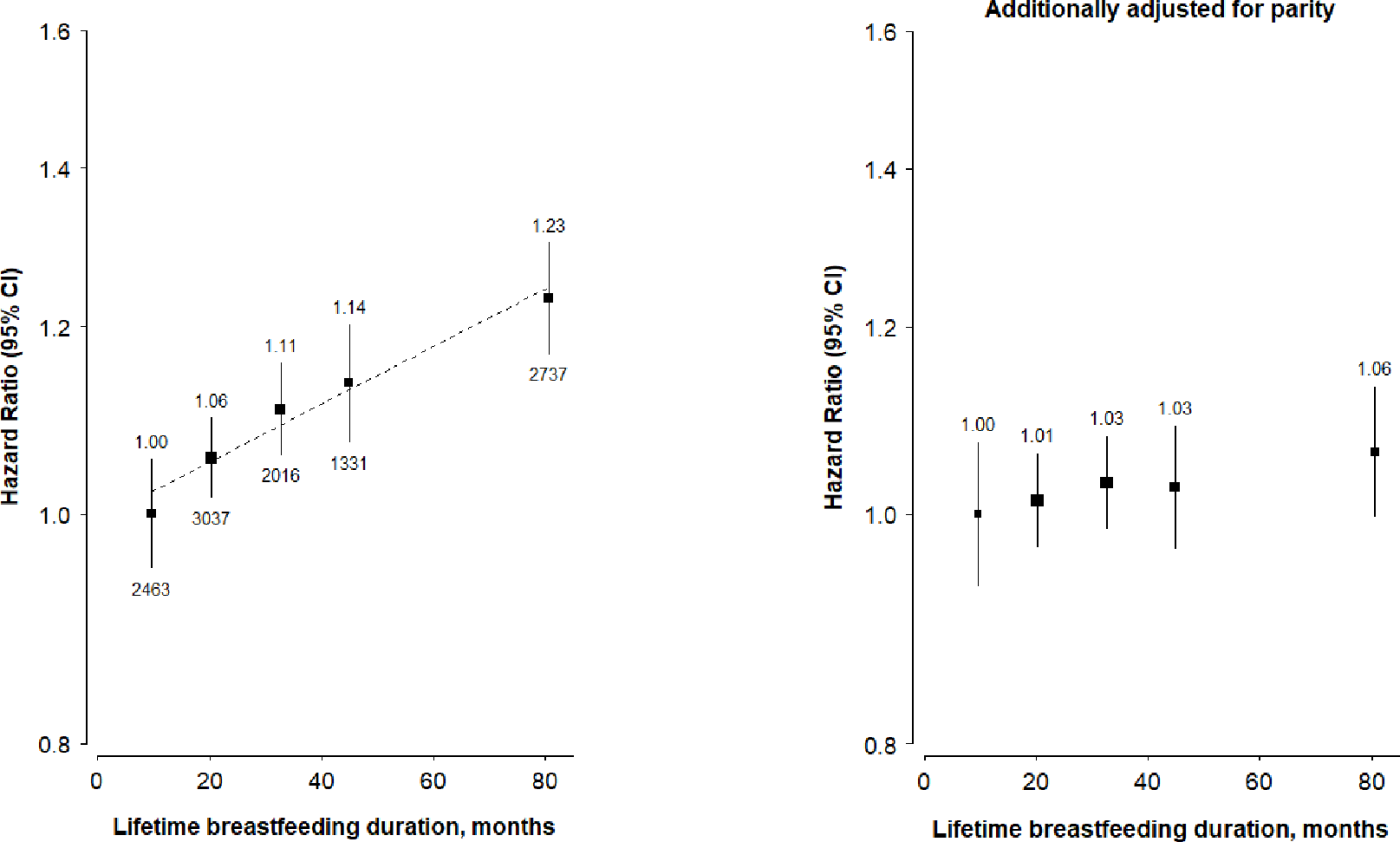
Association of lifetime breastfeeding duration with incident type 2 diabetes among ever breastfeeding parous women. Models were stratified by age-at-risk and study area and adjusted for education, household income, smoking status, alcohol drinking, physical activity, diet and family history of diabetes. The area of each square is inversely proportional to the SE of the log risk. Vertical lines indicate corresponding 95% CIs. The numbers above the squares are the HRs and the numbers below the squares are the number of type 2 diabetes diagnoses in that group.

During 3.3 million person-years (mean 11.8 years) of follow-up, 12 011 female participants (4.2%) were newly diagnosed with T2D. Compared with parous participants who never breastfed, mothers who ever breastfed had a similar risk of incident T2D (adjusted HR 1.06 (95% CI 0.94 to 1.20)). There was a modest log-linear positive association of lifetime breastfeeding duration with risk of incident T2D among parous participants who ever breastfed after adjustment for age, study area, socioeconomic position, lifestyle factors and family history of diabetes (figure 2). Compared with a lifetime breastfeeding duration of 1–12 months, breastfeeding durations of 13–24, 25–36, 37–48 and >48 months were associated with adjusted HRs of 1.06 (95% CI 1.02 to 1.10), 1.11 (1.06 to 1.16), 1.14 (1.07 to 1.20) and 1.23 (1.17 to 1.30), respectively (p value for trend <0.001), equivalent to a HR of 1.01 (1.01 to 1.02) per 6 months longer breastfeeding duration. However, after adjustment for parity (which was positively associated with the risk of incident T2D; online supplemental table S2), the association was fully attenuated (HR 1.00 (95% CI 0.99 to 1.01)) (figure 2). Similar findings were observed when nulliparous participants comprised the reference group and separate additional adjustment for BMI and waist circumference attenuated the association of lifetime breastfeeding duration with incident T2D only slightly (online supplemental table S2). Sensitivity analyses additionally adjusting for birth cohort did not materially alter the observed associations.

**Figure 3:**
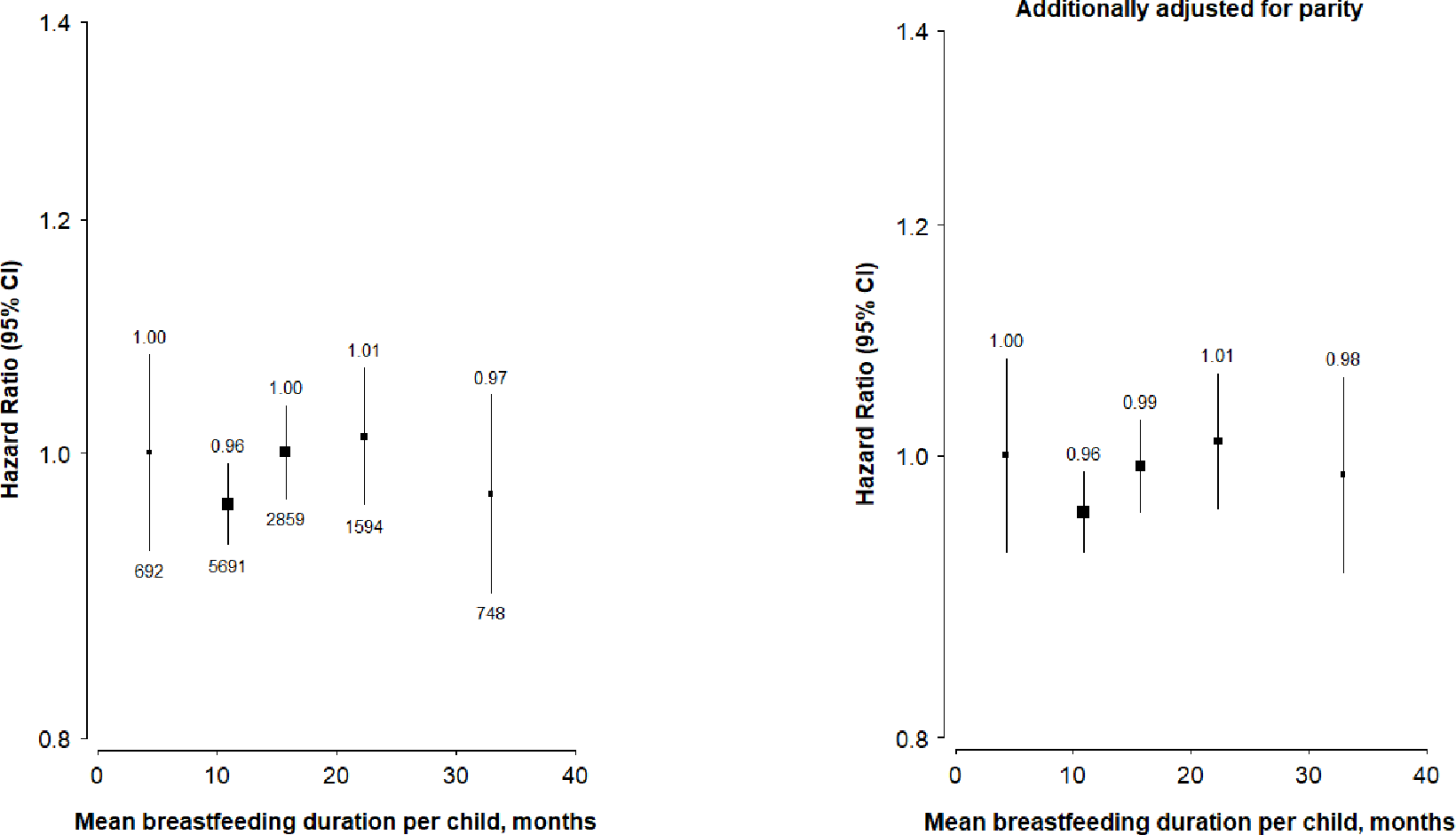
Association of mean breastfeeding duration per child with incident type 2 diabetes among ever breastfeeding parous women. Models were stratified by age-at-risk and study area and adjusted for education, household income, smoking status, alcohol drinking, physical activity, diet and family history of diabetes. The area of each square is inversely proportional to the SE of the log risk. Vertical lines indicate corresponding 95% CIs. The numbers above the squares are the HRs and the numbers below the squares are the number of type 2 diabetes diagnoses in that group.

After adjustment for age, study area, socioeconomic position, lifestyle factors and family history of diabetes, there was no apparent association of mean breastfeeding duration per child with risk of incident T2D (figure 3). Compared with a mean breastfeeding duration of 1–6 months per child, durations of 7–12, 13–18, 19–24 and >24 months per child were associated with adjusted HRs of 0.96 (95% CI 0.93 to 0.99), 0.99 (0.96 to 1.04), 1.01 (0.96 to 1.07) and 0.98 (0.90 to 1.05), respectively (p value for trend=0.39). The association was similar when the reference group comprised nulliparous participants (online supplemental table S2). Moreover, additional adjustment for BMI and waist circumference (online supplemental table S2) or birth cohort did not substantially alter the relationship.

The associations with incident T2D of lifetime breastfeeding duration and breastfeeding duration per child were similar in urban and rural areas (online supplemental figure S2), across birth cohorts (online supplemental figure S3) and across strata of BMI (online supplemental figure S4).

In analyses examining associations of additional reproductive factors, clear inverse associations with incident T2D were observed for age at menarche (p value for trend <0.001) and age at first birth (p value for trend <0.001) (online supplemental table S2). There were modest J-shaped associations of reproductive years and age at menopause with risk of incident T2D. Adjustment of the associations of lifetime breastfeeding duration and mean breastfeeding duration per child for these further reproductive factors, in addition to parity, did not markedly alter the associations (online supplemental figures S5 and S6). Furthermore, the relationships differed little across strata of these and additional reproductive factors (online supplemental figures S7–S10).

## Conclusions

In this large prospective cohort study including >280 000 women from urban and rural regions of China with almost universal childbearing and breastfeeding, there was no apparent association of breastfeeding with risk of maternal incident T2D. Although a modest positive relationship was observed between lifetime breastfeeding duration and T2D risk, this disappeared completely following adjustment for parity, consistent with the absence of an association of mean breastfeeding duration per child with maternal T2D risk.

Our study showed comparable incident T2D risks among ever and never breastfeeding parous female participants after controlling for age, study area, socioeconomic position, lifestyle factors and family history of diabetes. This differs from the findings of a meta-analysis of seven prospective studies, including approximately 340 000 women, which reported 27% lower risk of maternal incident T2D (relative risk (RR) 0.73 (95% CI 0.68 to 0.79)) associated with ever versus never breastfeeding.^6^ However, these findings were dominated by one large retrospective cohort study based on administrative data, which was unable to control for potentially important confounders, including lifestyle factors.^22^ In contrast, but consistent with our findings, analyses from the US Nurses’ Health Studies I and II showed null associations of ever versus never breastfeeding with risk of maternal incident T2D; after controlling for age, parity, BMI at 18 years, lifestyle factors and family history of diabetes, these two prospective cohort studies, including 121 700 and 116 671 women, respectively, showed RRs of 0.97 (95% CI 0.91 to 1.02) and 0.90 (0.77 to 1.04).^5^ A similar finding was observed in the Shanghai Women’s Health Study, the only previous prospective study examining this relationship in China.^16^ Including 63 000 parous women, of whom 1561 developed incident T2D during 4.6 years of follow-up, this study observed no clear difference in T2D risk between never and ever breastfeeding women (RR 0.88 (95% CI 0.76 to 1.02)) after adjustment for age, socioeconomic status, lifestyle factors, adiposity, parity and hypertension.^16^

In the present study, we observed a modest positive log-linear relationship between lifetime breastfeeding duration and risk of incident T2D, with each 6 months longer breastfeeding associated with 1% higher risk. This is in contrast with several previous studies, which have tended to show modest inverse associations. For example, a dose-response meta-analysis of data from nine prospective studies, eight of which were conducted in high-income countries including the USA, Canada, Australia and European countries, suggested that each month longer breastfeeding was associated with a modest 1% lower risk of T2D (RR 0.991 (95% CI 0.984 to 0.998)).^6^ However, this was with the caveats that the overall quality of included studies was modest, levels of adjustment varied and there was evidence of publication bias.^6^ Nevertheless, reasonably consistent inverse associations were observed in the Nurses’ Health Studies, in which each additional year of breastfeeding over a woman’s lifetime was associated with approximately 15% lower T2D risk (HR 0.85 (95% CI 0.73 to 0.99) and 0.86 (0.79–0.93) in Nurses’ Health Study I and II, respectively), independent of age, lifestyle factors, BMI at 18 years, participant birthweight, family history of diabetes, multivitamin use and parity.^5^ However, consistent with our findings, in the Shanghai Women’s Health Study, when compared with parous women who never breastfed and after controlling for age, socioeconomic position, lifestyle factors and hypertension, there was no clear association of lifetime breastfeeding duration with risk of T2D (p value for trend=0.75), with a lower risk observed only in the longest breastfeeding duration category (≥36 months: HR 0.75 (95% CI 0.61 to 0.92)) after additional adjustment for adiposity.^16^ This association remained unchanged following additional adjustment for parity. Of note, lifetime breastfeeding durations were typically longer in the present study and the Shanghai Women’s Health Study (12% and 35%^16^ of parous women, respectively, reported lifetime breastfeeding durations of less than 6 months) than in the Nurses’ Health Studies (55%).^23^

The discordance of study findings could, at least in part, be attributed to differences in breastfeeding practices, patterns and correlates between and within populations. Strong positive relationships are typically seen between measures of socioeconomic position and breastfeeding initiation and duration in high-income countries.^24^,^29^ However, in many low-income and middle-income countries, breastfeeding is typically inversely associated with socioeconomic position,^30 31^ as observed in the present and other Chinese study populations.^16^ Given the relevance of socioeconomic position for T2D risk,^32 33^ this may contribute to the apparent differences in the relevance of breastfeeding for T2D risk between these populations. Furthermore, correlations between breastfeeding and adiposity—the major modifiable and causal risk factor for T2D^3 34^—differ between populations. An inverse dose-response relationship has typically been described in high-income settings,^35^ in contrast with the J-shaped association observed in the present and other studies in China.^16^ Given likely errors in measurement of these T2D risk factors (in particular socioeconomic position^36^), and the very strong association of adiposity with T2D risk,^3 34^ residual confounding is expected. This could underlie differences between populations in the associations of breastfeeding with maternal T2D risk and could explain the modest apparent protective effects of breastfeeding observed in previous studies.

We found no association between breastfeeding duration per child and risk of incident T2D in CKB, and the log-linear positive association between lifetime breastfeeding duration and T2D was fully attenuated after adjustment for parity. This is consistent with previously described positive associations of parity with T2D risk,^15 37 38^ including in CKB.^39^ However, as reported previously,^39^ a strikingly similar relationship has been observed between number of children and T2D risk in women and men (online supplemental figure S11). This provides support for the relevance of non-biological factors, including socioeconomic position but also potentially cultural and lifestyle influences (eg, reduced levels of physical activity or increased intake of less healthy foods among those with larger families^39^), in contributing to the association of parity and, in turn, of breastfeeding with T2D risk.

Apart from the large study population and availability of detailed information on a range of reproductive, sociodemographic and lifestyle‐related factors, our study has several strengths. The opportunity to investigate the relationship of breastfeeding with T2D in an East Asian population provides unique insights into the nature and independence of the association. Almost universal breastfeeding among parous women in CKB provided enhanced statistical power to examine the relevance of breastfeeding duration. Review of the medical records of almost 1000 participants who developed incident diabetes during follow-up confirmed the validity of the diagnosis (positive predictive value 97%, based on American Diabetes Association diagnostic criteria^40^ and medication use). Finally, inclusion of women from 10 diverse regions and minimal loss to follow-up enhanced generalisability of the findings. However, our study has certain limitations. A proportion of incident T2D cases will have remained undiagnosed in the study population, likely disproportionately affecting women in lower socioeconomic groups. However, any bias resulting from this misclassification of T2D status would be expected to result in lower risks of incident T2D associated with ever versus never breastfeeding and with longer breastfeeding duration, which were not observed in the present study. Given the age of the cohort, all incident diabetes cases were assumed to be T2D; however, a small proportion may have been type 1 diabetes. Women with previously diagnosed or screen-detected diabetes at recruitment were excluded from the present analyses which may have included some women who developed T2D after completion of breastfeeding. Furthermore, data were not available on history of gestational diabetes at baseline, which may modify the association of breastfeeding with risk of incident T2D. However, in a 2020 resurvey of a randomly selected sample of approximately 5% of surviving CKB participants, less than 0.2% of parous women reported a history of gestational diabetes. The high frequency of breastfeeding precluded investigation of the relevance of breastfeeding initiation among population subgroups. Moreover, the small proportion of women who reported lifetime breastfeeding durations of less than 6 months prevented detailed investigation of the relevance of such durations for risk of T2D, and differing associations at these shorter durations cannot be excluded based on the presented findings. However, the typically longer durations of breastfeeding in the present study population, when compared with Western populations, allowed exploration of a wider distribution of breastfeeding duration (up to approximately 6 years). Furthermore, akin to most previous studies, information on breastfeeding was self‐reported and obtained several years after childbirth, potentially contributing to measurement error.

This large prospective cohort study among women in China offers valuable insights into the relevance of breastfeeding for maternal T2D risk. After controlling for key confounders, including parity, in this population in which most women were parous and reported breastfeeding for relatively long durations, we found no association of breastfeeding initiation or duration with incident T2D. This contrasts with modest inverse associations in previous, predominantly Western population, studies. The divergent findings conceivably reflect the associations with T2D of social, economic and lifestyle correlates of breastfeeding, which differ between populations. While the health advantages of breastfeeding for both mother and child are clear, our findings suggest likely limited relevance for maternal T2D prevention.

## Supplementary material

10.1136/bmjopen-2025-109377 online supplemental file 1

## Data Availability

Data are available upon reasonable request.
