# Supplementary material for: Breastfeeding and risk of maternal type 2 diabetes: a prospective cohort study of 280 000 women in China
Source: BMJ Open. 2026 Jun 28;16(6):e109377. doi: 10.1136/bmjopen-2025-109377 (PMC13311600; doi:10.1136/bmjopen-2025-109377)

Breastfeeding and risk of maternal type 2 diabetes: a prospective cohort study of 280 000 women in China

Online Supplemental Material, Table of Contents

	**Page**
Members of the China Kadoorie Biobank collaborative group	2
Supplemental Table S1. Baseline characteristics of study participants by mean breastfeeding duration per child	3
Supplemental Table S2. Association of reproductive factors with incident type 2 diabetes	4
Supplemental Figure S1. Locations of the China Kadoorie Biobank recruitment centres	5
Supplemental Figure S2. Association of mean breastfeeding duration per child and lifetime breastfeeding duration with incident type 2 diabetes among ever breastfeeding parous women in urban and rural areas	6
Supplemental Figure S3. Association of mean breastfeeding duration per child and lifetime breastfeeding duration with incident type 2 diabetes among ever breastfeeding parous women, by birth cohort	7
Supplemental Figure S4. Association of mean breastfeeding duration per child and lifetime breastfeeding duration with incident type 2 diabetes among ever breastfeeding parous women, by BMI	8
Supplemental Figure S5. Association of lifetime breastfeeding duration with incident type 2 diabetes among ever breastfeeding parous women	9
Supplemental Figure S6. Association of mean breastfeeding duration per child with incident type 2 diabetes among ever breastfeeding parous women	10
Supplemental Figure S7. Association of mean breastfeeding duration per child and lifetime breastfeeding duration with incident type 2 diabetes among ever breastfeeding parous women, by number of live births	11
Supplemental Figure S8. Association of mean breastfeeding duration per child and lifetime breastfeeding duration with incident type 2 diabetes among ever breastfeeding parous women, by age at menarche	12
Supplemental Figure S9. Association of mean breastfeeding duration per child and lifetime breastfeeding duration with incident type 2 diabetes among ever breastfeeding parous women, by age at first birth	13
Supplemental Figure S10. Association of mean breastfeeding duration per child and lifetime breastfeeding duration with incident type 2 diabetes among ever breastfeeding parous women, by menopause status	14
Supplemental Figure S11. Association of number of children with incident type 2 diabetes in men and women	15

Members of the China Kadoorie Biobank collaborative group:

International Steering Committee: Junshi Chen, Zhengming Chen (PI), Robert Clarke, Rory Collins, Liming Li (PI), Jun Lv, Richard Peto, Robin Walters.

International Co-ordinating Centre, Oxford: Daniel Avery, Maxim Barnard, Derrick Bennett, Ruth Boxall, Ka Hung Chan, Yiping Chen, Zhengming Chen, Charlotte Clarke, Jonathan Clarke; Robert Clarke, Huaidong Du, Geoffrey Ma, Ahmed Edris Mohamed, Hannah Fry, Simon Gilbert, Pek Kei Im, Andri Iona, Maria Kakkoura, Christiana Kartsonaki, Hubert Lam, Kuang Lin, James Liu, Mohsen Mazidi, Iona Millwood, Sam Morris, Qunhua Nie, Alfred Pozarickij, Maryam Rahmati, Paul Ryder, Dan Schmidt, Becky Stevens, Iain Turnbull, Robin Walters, Baihan Wang, Lin Wang, Neil Wright, Ling Yang, Xiaoming Yang, Pang Yao.

National Co-ordinating Centre, Beijing: Xiao Han, Can Hou, Qingmei Xia, Chao Liu, Jun Lv, Pei Pei, Dianjianyi Sun, Canqing Yu, Lang Pan.

10 Regional Co-ordinating Centres: Qingdao CDC: Zengchang Pang, Ruqin Gao, Shanpeng Li, Haiping Duan, Shaojie Wang, Yongmei Liu, Ranran Du, Yajing Zang, Liang Cheng, Xiaocao Tian, Hua Zhang, Yaoming Zhai, Feng Ning, Xiaohui Sun, Feifei Li. **Licang CDC:** Silu Lv, Junzheng Wang, Wei Hou. **Heilongjiang Provincial CDC:** Wei Sun, Shichun Yan, Xiaoming Cui. **Nangang CDC:** Chi Wang, Zhenyuan Wu,Yanjie Li, Quan Kang. **Hainan Provincial CDC:** Huiming Luo, Tingting Ou. **Meilan CDC:** Xiangyang Zheng, Zhendong Guo, Shukuan Wu, Yilei Li, Huimei Li. **Jiangsu Provincial CDC:** Ming Wu, Yonglin Zhou, Jinyi Zhou, Ran Tao, Jie Yang, Jian Su. **Suzhou CDC:** Fang Liu, Jun Zhang, Yihe Hu, Yan Lu, Liangcai Ma, Aiyu Tang, Shuo Zhang, Jianrong Jin, Jingchao Liu. **Guangxi Provincial CDC:** Mei Lin, Zhenzhen Lu. **Liuzhou CDC:** Lifang Zhou, Changping Xie, Jian Lan,Tingping Zhu,Yun Liu, Liuping Wei, Liyuan Zhou, Ningyu Chen, Yulu Qin, Sisi Wang. **Sichuan Provincial CDC:** Xianping Wu, Ningmei Zhang, Xiaofang Chen, Xiaoyu Chang. **Pengzhou CDC:** Mingqiang Yuan, Xia Wu, Xiaofang Chen, Wei Jiang, Jiaqiu Liu, Qiang Sun. **Gansu Provincial CDC:** Faqing Chen, Xiaolan Ren, Caixia Dong. **Maiji CDC:** Hui Zhang, Enke Mao, Xiaoping Wang, Tao Wang, Xi zhang. **Henan Provincial CDC:** Kai Kang, Shixian Feng, Huizi Tian, Lei Fan. **Huixian CDC:** XiaoLin Li, Huarong Sun, Pan He, Xukui Zhang. **Zhejiang Provincial CDC:** Min Yu, Ruying Hu, Hao Wang. **Tongxiang CDC**: Xiaoyi Zhang, Yuan Cao, Kaixu Xie, Lingli Chen, Dun Shen. **Hunan Provincial CDC:** Xiaojun Li, Donghui Jin, Li Yin, Huilin Liu, Zhongxi Fu. **Liuyang CDC:** Xin Xu, Hao Zhang, Jianwei Chen,Yuan Peng, Libo Zhang, Chan Qu.

Supplemental Table S1. Baseline characteristics of study participants by mean breastfeeding duration per child

Characteristic*	Nulliparous	Parous women						
		Never breastfed	Mean breastfeeding duration per child, months					
			<7	7-12	13-18	19-24	>24	Total
No. of participants	3852	7724	17,600	128,353	64,698	41,916	19,712	272,279
**Age and socioeconomic factors**								
Mean age (SD), y	51.0 (11.7)	46.8 (9.4)	46.8 (9.5)	49.6 (10.2)	51.9 (10.5)	56.3 (10.8)	58.1 (9.8)	51.1 (10.4)
Living in rural area, %	37.7	28.3	36.2	43.9	67.5	80.6	85.2	57.4
6+ years of education, %	46.8	55.6	52.1	45.6	41.9	35.6	31.4	43.5
Annual household income ≥20,000 yuan, %	34.1	45.3	45.3	41.8	39.9	35.5	33.6	40.6
**Lifestyle factors**								
Ever smoker, %	5.8	4.2	4.5	4.6	5.0	5.9	7.1	4.9
Ever regular alcohol drinker, %	3.6	2.7	3.2	2.8	3.0	3.4	3.9	3.0
Mean physical activity (SD), MET-h/d	19.8 (11.7)	18.9 (12.0)	20.2 (12.7)	20.5 (13.2)	21.1 (12.4)	20.9 (12.8)	21.1 (12.4)	20.8 (12.8)
Regular consumption†, %								
Fresh fruit	31.7	38.4	36.1	32.1	31.9	29.1	28.7	31.8
Meat	39.9	44.1	45.5	44.4	43.5	42.0	42.7	44.0
Soybean products	9.6	11.1	11.3	9.7	8.2	7.4	9.3	9.1
Rice	71.6	73.2	72.9	72.6	72.5	72.2	72.9	72.4
Wheat	46.0	48.2	47.2	44.9	44.4	44.8	44.5	44.7
**Anthropometry, blood pressure and plasma glucose, mean (SD)**								
BMI, kg/m^2	23.6 (3.7)	23.5 (3.4)	23.5 (3.3)	23.7 (3.4)	23.9 (3.5)	24.0 (3.5)	24.0 (3.6)	23.7 (3.4)
WC, cm	78 (10)	78 (9)	78 (9)	79 (9)	79 (10)	79 (10)	79 (10)	79 (9)
RPG, mmol/L	5.8 (1.1)	5.7 (1.0)	5.7 (1.1)	5.7 (1.1)	5.7 (1.1)	5.8 (1.2)	5.7 (1.2)	5.7 (1.1)
SBP, mmHg	130 (23)	128 (20)	128 (20)	129 (21)	129 (22)	130 (23)	131 (23)	129 (22)
**Reproductive history**								
Mean age at menarche (SD), y	15.5 (2.6)	14.9 (1.9)	15.0 (1.9)	15.3 (1.9)	15.5 (1.9)	15.9 (2.0)	16.2 (2.0)	15.5 (2.0)
Mean number of live births (SD)	NA	1.8 (0.9)	2.1 (1.0)	2.3 (1.2)	2.3 (1.4)	2.3 (1.4)	2.2 (1.2)	2.2 (1.3)
Mean age at first birth (SD), y	NA	25.1 (3.8)	24.2 (3.4)	23.5 (3.2)	23.1 (3.0)	22.6 (2.9)	22.4 (2.8)	23.4 (3.1)
History of miscarriage, %	39.1	10.6	9.1	8.9	9.2	9.6	9.9	8.9
History of induced abortion, %	54.0	52.7	56.2	53.1	52.5	50.2	49.0	52.4
History of stillbirth, %	19.4	6.3	5.2	5.2	5.6	6.4	6.6	5.6
Mean lifetime breastfeeding duration (SD), months	NA	NA	9.1 (5.7)	24.7 (13.9)	35.6 (21.9)	50.1 (29.8)	69.0 (38.0)	34.8 (29.4)
Mean breastfeeding duration per child (SD), months	NA	NA	4.3 (1.8)	10.9 (1.5)	15.5 (2.0)	22.4 (1.9)	32.9 (7.5)	14.9 (7.3)
Mean age at menopause‡ (SD), y	45.2 (7.1)	46.8 (4.8)	47.5 (4.5)	48.0 (4.3)	48.3 (4.4)	48.4 (4.4)	48.4 (4.3)	48.2 (4.4)
**Medical history, %**								
Cancer	0.8	0.6	0.8	0.4	0.5	0.5	0.4	0.5
Stroke/TIA	1.1	1.0	1.1	1.1	1.1	1.1	1.2	1.1
Coronary heart disease	2.6	4.0	3.4	2.8	3.0	2.5	2.7	2.8
Chronic kidney disease	1.6	2.1	1.9	1.6	1.5	1.4	1.5	1.6
Poor self-rated health	13.3	15.7	12.8	10.4	10.4	11.1	14.2	10.5
Family history of diabetes	7.8	8.4	7.5	6.5	6.4	5.4	5.5	6.3

*Standardised, where appropriate to age, and study area structure of the study population; †Defined as ≥4 days per week; ‡Among 144,069 post-menopausal women.
BMI=body mass index; MET-h/d=metabolic equivalent of task hours per day; RPG=random plasma glucose; SBP=systolic blood pressure; TIA=transient ischaemic attack; WC, waist circumference.

Supplemental Table S2. Association of reproductive factors with incident type 2 diabetes

Reproductive factor	No. of events	HR (95% CI)	
		Model A	Model B
**Lifetime breastfeeding duration**			
Nulliparous	143	1.00 (0.84-1.19)	1.00 (0.84-1.19)
Never breastfed	284	0.92 (0.81-1.03)	0.92 (0.81-1.03)
0-12 months	2463	0.92 (0.88-0.97)	0.92 (0.88-0.97)
13-24 months	3037	0.97 (0.94-1.01)	0.97 (0.94-1.01)
25-36 months	2016	1.02 (0.97-1.07)	1.02 (0.97-1.07)
37-48 months	1331	1.04 (0.98-1.10)	1.04 (0.98-1.10)
>48 months	2737	1.13 (1.07-1.19)	1.13 (1.07-1.19)
		Trend: χ2=30.7 (*p*<0.001)	*Trend*: χ2=13.4 (*p*<0.001)
**Mean breastfeeding duration per child**			
Nulliparous	143	1.00 (0.84-1.19)	1.00 (0.84-1.19)
Never breastfed	284	0.94 (0.84-1.06)	0.94 (0.83-1.06)
0-6 months	692	1.02 (0.95-1.10)	1.04 (0.96-1.12)
7-12 months	5691	0.98 (0.95-1.01)	0.96 (0.93-0.99)
13-18 months	2859	1.02 (0.99-1.06)	0.97 (0.94-1.01)
19-24 months	1594	1.04 (0.98-1.09)	1.00(0.95-1.06)
>24 months	748	0.99 (0.92-1.07)	0.96 (0.89-1.04)
		Trend: χ2=1.33 (*p*=0.25)	*Trend*: χ2=0.05 (*p*=0.82)
**No. of live births**			
0	143	1.00 (0.84-1.19)	1.00 (0.84-1.19)
1	3454	0.90 (0.85-0.95)	0.90 (0.85-0.95)
2	3784	0.99 (0.96-1.03)	0.95 (0.91-0.98)
3	2378	1.06 (1.01-1.11)	1.01 (0.97-1.06)
4	1235	1.16 (1.08-1.23)	1.08 (1.01-1.15)
>4	1017	1.34 (1.23-1.45)	1.21 (1.11-1.32)
		Trend: χ2=72.7 (*p*<0.001)	*Trend*: χ2=40.8 (*p*<0.001)
**Age at menarche, y**			
≤13	1909	1.00 (0.95-1.05)	1.00 (0.95-1.05)
>13 and ≤14	1747	0.88 (0.84-0.93)	0.93 (0.89-0.98)
>14 and ≤15	2239	0.88 (0.85-0.92)	0.96 (0.92-1.00)
>15 and ≤16	2336	0.85 (0.81-0.88)	0.95 (0.91-0.99)
>16 and ≤18	3046	0.77 (0.74-0.80)	0.90 (0.87-0.94)
>18	734	0.69 (0.64-0.75)	0.88 (0.81-0.94)
		Trend: χ2=96.2 (*p*<0.001)	*Trend*: χ2=11.0 (*p*<0.001)
**Age at first birth, y**			
≤20	3109	1.00 (0.96-1.04)	1.00 (0.96-1.04)
>20 and ≤22	2584	0.82 (0.79-0.86)	0.88 (0.84-0.92)
>22 and ≤24	2803	0.78 (0.75-0.81)	0.86 (0.83-0.90)
>24 and ≤29	3028	0.71 (0.68-0.75)	0.82 (0.79-0.86)
>29	344	0.67 (0.60-0.75)	0.78 (0.70-0.87)
		Trend: χ2=126.2 (*p*<0.001)	*Trend*: χ2=41.4 (*p*<0.001)
**Age at menopause, y**			
≤42	798	1.00 (0.93-1.08)	1.00 (0.93-1.08)
>42 and ≤45	854	0.95 (0.89-1.02)	0.96 (0.90-1.03)
>45 and ≤48	1952	0.95 (0.91-1.00)	0.95 (0.91-0.99)
>48 and ≤50	1943	0.98 (0.94-1.03)	0.97 (0.93-1.02)
>50 and ≤52	1369	1.05 (1.00-1.11)	1.00 (0.95-1.06)
>52	1166	1.08 (1.01-1.14)	0.99 (0.94-1.06)
		Trend: χ2=9.2 (*p*=0.003)	*Trend*: χ2=0.61 (*p*=0.4)
**No. of reproductive years**			
≤26	809	1.00 (0.93-1.08)	1.00 (0.93-1.08)
>26 and ≤29	828	0.92 (0.86 - 0.99)	0.92 (0.85-0.98)
>29 and ≤32	2009	0.97 (0.93-1.02)	0.95 (0.91-1.00)
>32 and ≤34	1525	0.99 (0.94-1.05)	0.94 (0.89-0.99)
>34 and ≤36	1369	1.08 (1.02-1.14)	0.99 (0.93-1.04)
>36 and ≤38	866	1.12 (1.04-1.20)	0.98 (0.91-1.05)
>38	676	1.33 (1.23-1.43)	1.13 (1.04-1.22)
		Trend: χ2=48.0 (*p*<0.001)	*Trend*: χ2=6.6 (*p*=0.01)

Model A: Models were stratified by age at risk and study area and adjusted for level of attained education, household income, smoking status, alcohol drinking, diet (including rice, wheat, meat, soybean and fresh fruit consumption) and family history of diabetes; Model B: Model A additionally adjusted for body mass index and waist circumference.

Supplemental Figure S1. Locations of the China Kadoorie Biobank recruitment centres

The South China Islands have been omitted for illustrative purposes

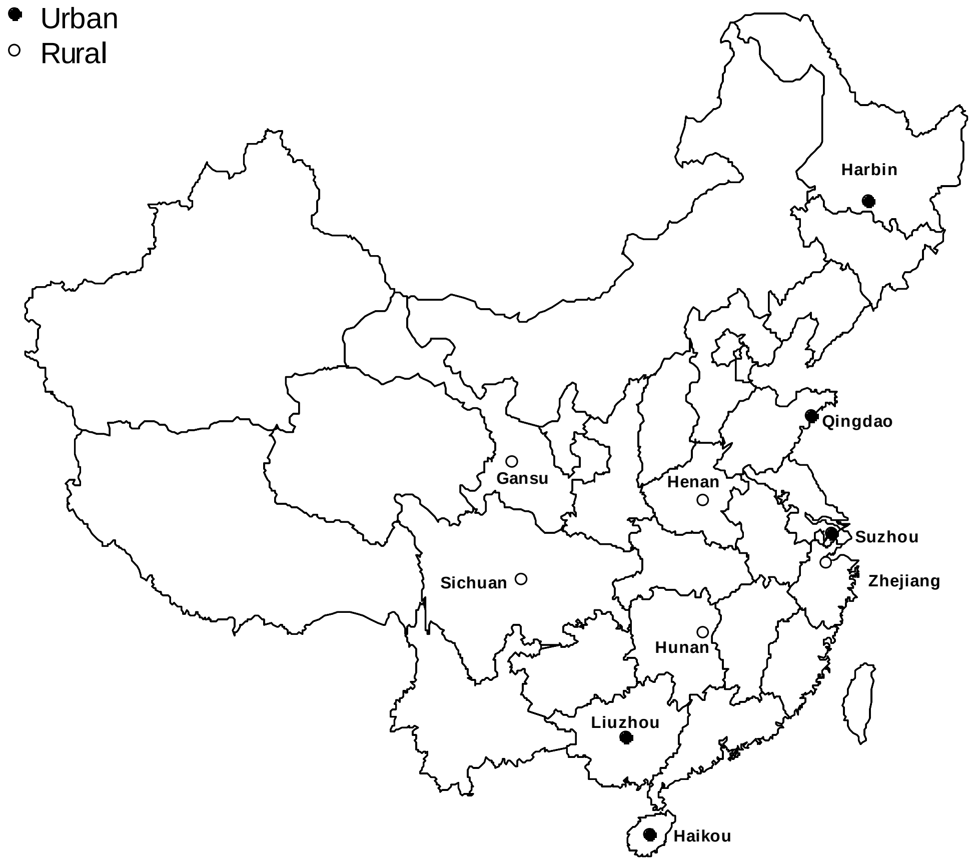

Supplemental Figure S2. Association of mean breastfeeding duration per child and lifetime breastfeeding duration with incident type 2 diabetes among ever breastfeeding parous women in urban and rural areas

Models were stratified by age-at-risk and study area and adjusted for education, household income, smoking status, alcohol drinking, physical activity, diet, and family history of diabetes. The area of each square is inversely proportional to the SE of the log risk. Vertical lines indicate corresponding 95% confidence intervals (CI). The numbers above the squares are the HRs and the numbers below the squares are the number of type 2 diabetes diagnoses in that group.

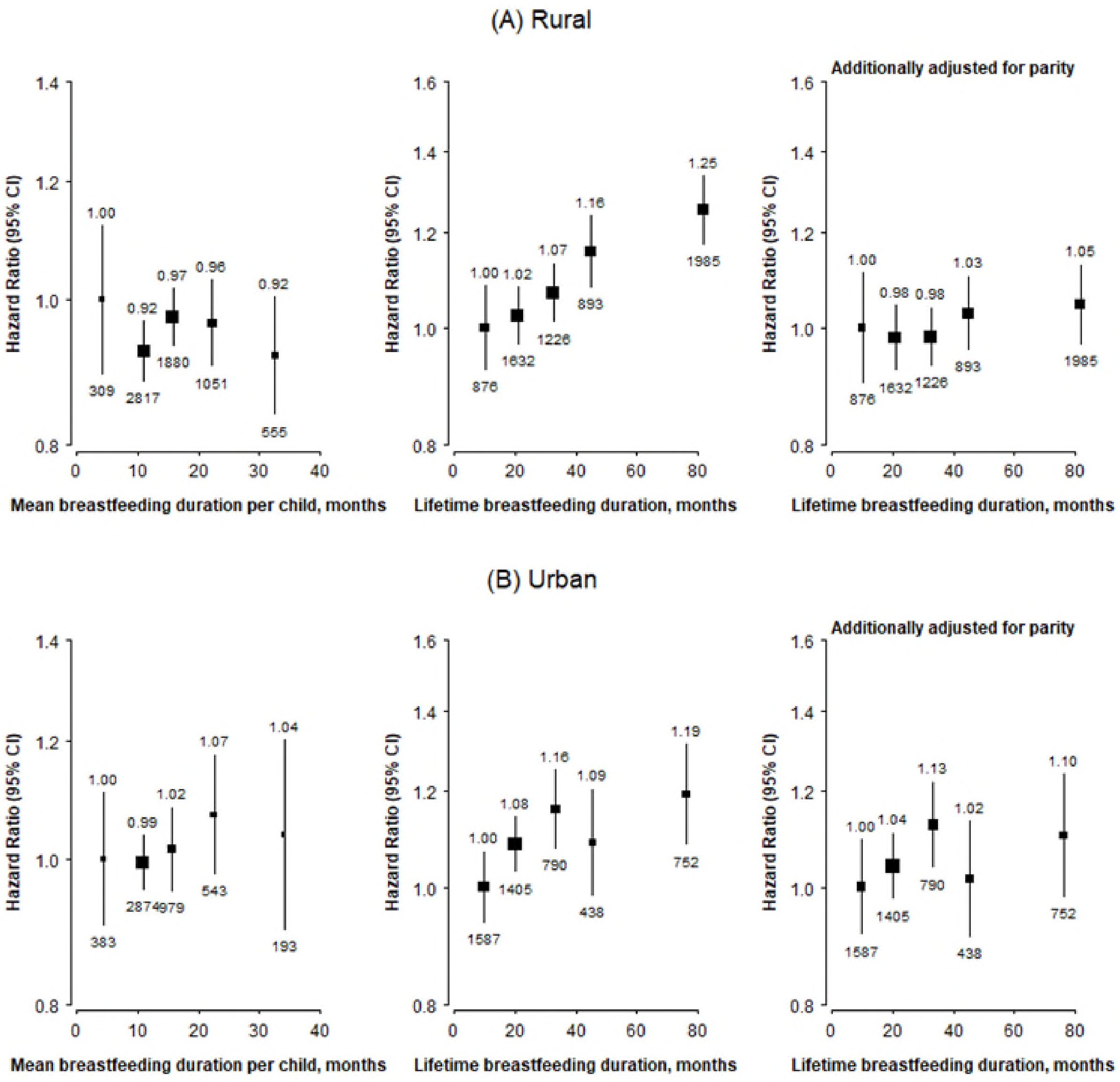

Supplemental Figure S3. Association of mean breastfeeding duration per child and lifetime breastfeeding duration with incident type 2 diabetes among ever breastfeeding parous women, by birth cohort

Models were stratified by age-at-risk and study area and adjusted for education, household income, smoking status, alcohol drinking, physical activity, diet, and family history of diabetes. The area of each square is inversely proportional to the SE of the log risk. Vertical lines indicate corresponding 95% confidence intervals (CI). The numbers above the squares are the HRs and the numbers below the squares are the number of type 2 diabetes diagnoses in that group.

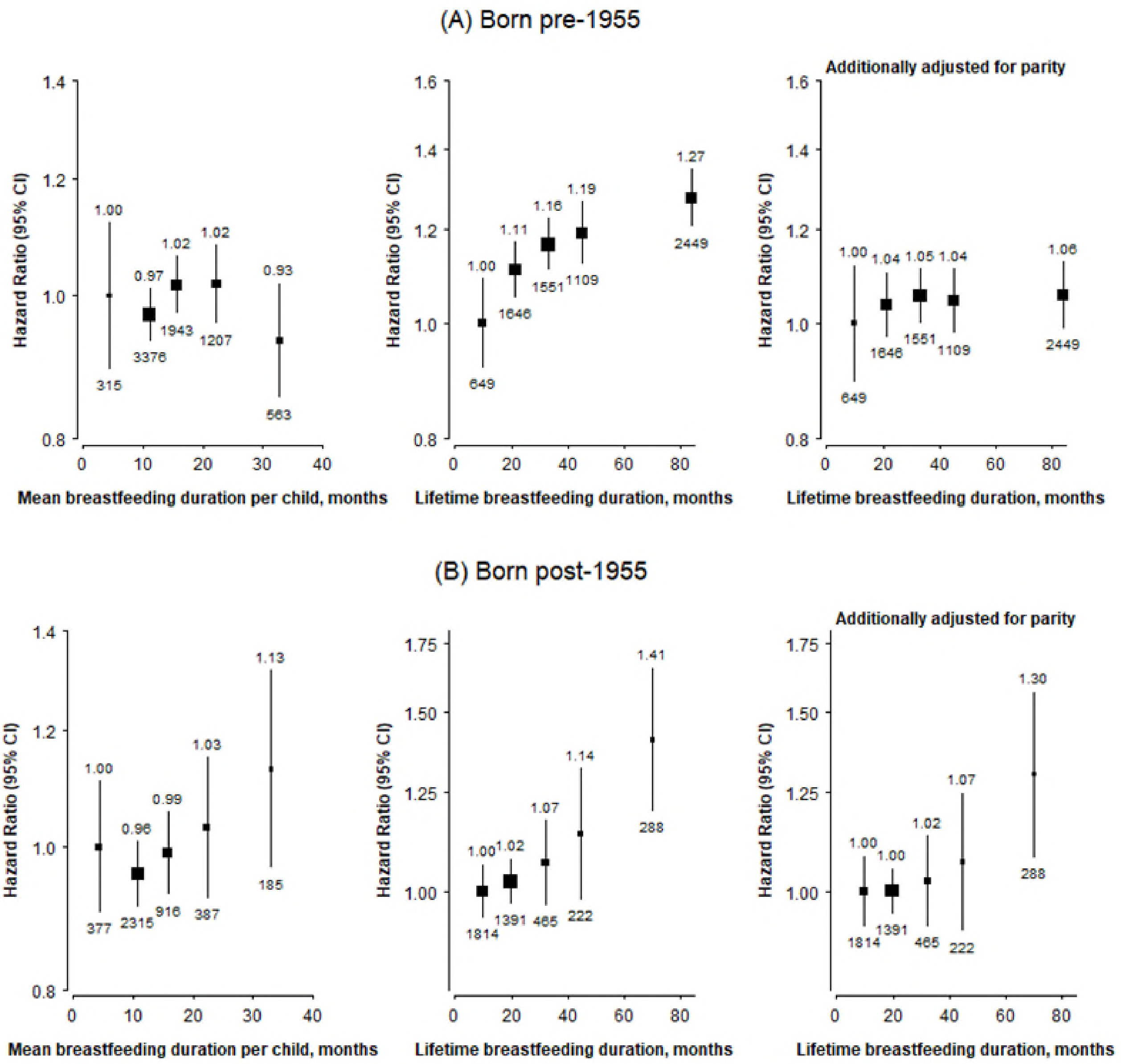

Supplemental Figure S4. Association of mean breastfeeding duration per child and lifetime breastfeeding duration with incident type 2 diabetes among ever breastfeeding parous women, by BMI

Models were stratified by age-at-risk and study area and adjusted for education, household income, smoking status, alcohol drinking, physical activity, diet, and family history of diabetes. The area of each square is inversely proportional to the SE of the log risk. Vertical lines indicate corresponding 95% confidence intervals (CI). The numbers above the squares are the HRs and the numbers below the squares are the number of type 2 diabetes diagnoses in that group.

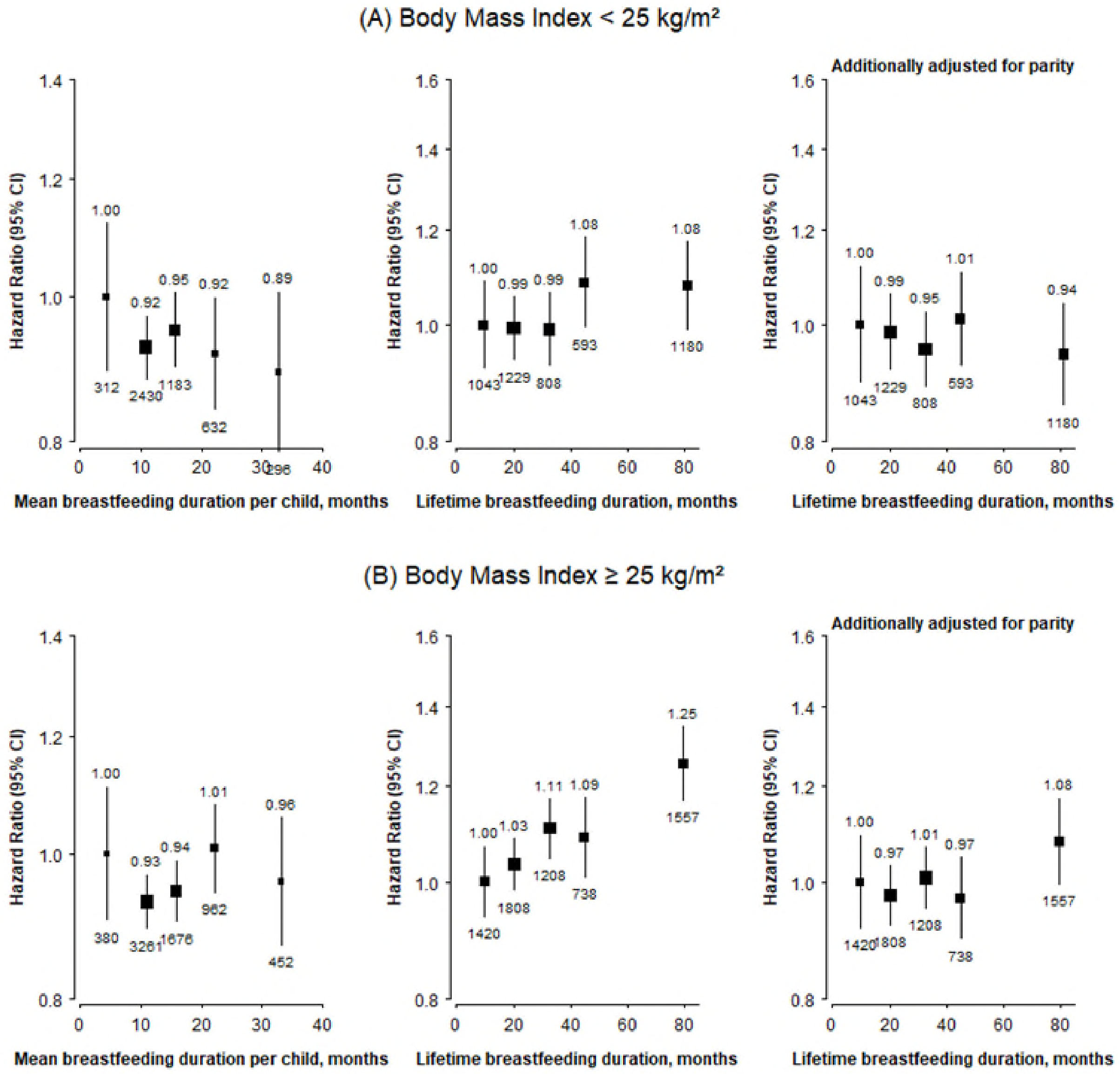

Supplemental Figure S5. Association of lifetime breastfeeding duration with incident type 2 diabetes among ever breastfeeding parous women

Models were stratified by age-at-risk and study area and adjusted for education, household income, smoking status, alcohol drinking, physical activity, diet, and family history of diabetes. The area of each square is inversely proportional to the SE of the log risk. Vertical lines indicate corresponding 95% confidence intervals (CI). The numbers above the squares are the HRs and the numbers below the squares are the number of type 2 diabetes diagnoses in that group.

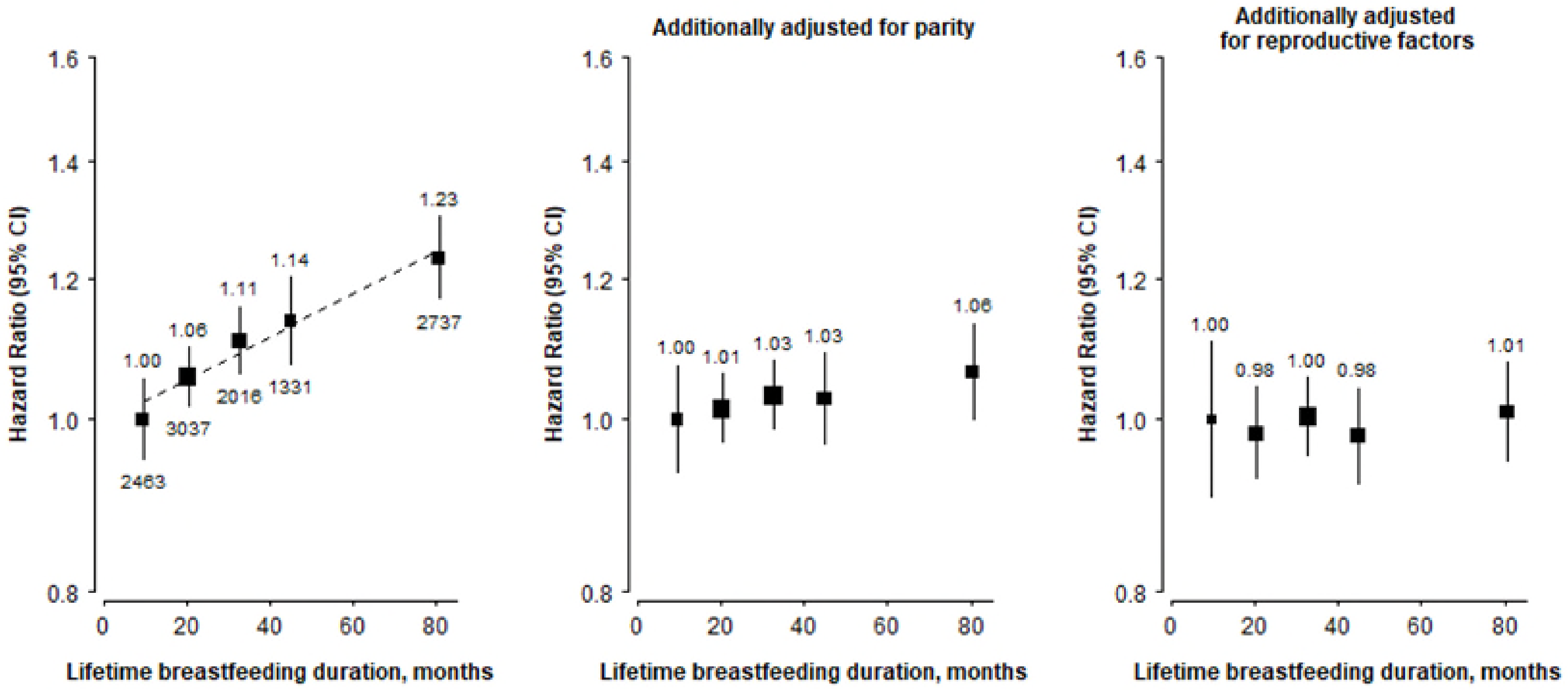

Supplemental Figure S6. Association of mean breastfeeding duration per child with incident type 2 diabetes among ever breastfeeding parous women

Models were stratified by age-at-risk and study area and adjusted for education, household income, smoking status, alcohol drinking, physical activity, diet, and family history of diabetes. The area of each square is inversely proportional to the SE of the log risk. Vertical lines indicate corresponding 95% confidence intervals (CI). The numbers above the squares are the HRs and the numbers below the squares are the number of type 2 diabetes diagnoses in that group.

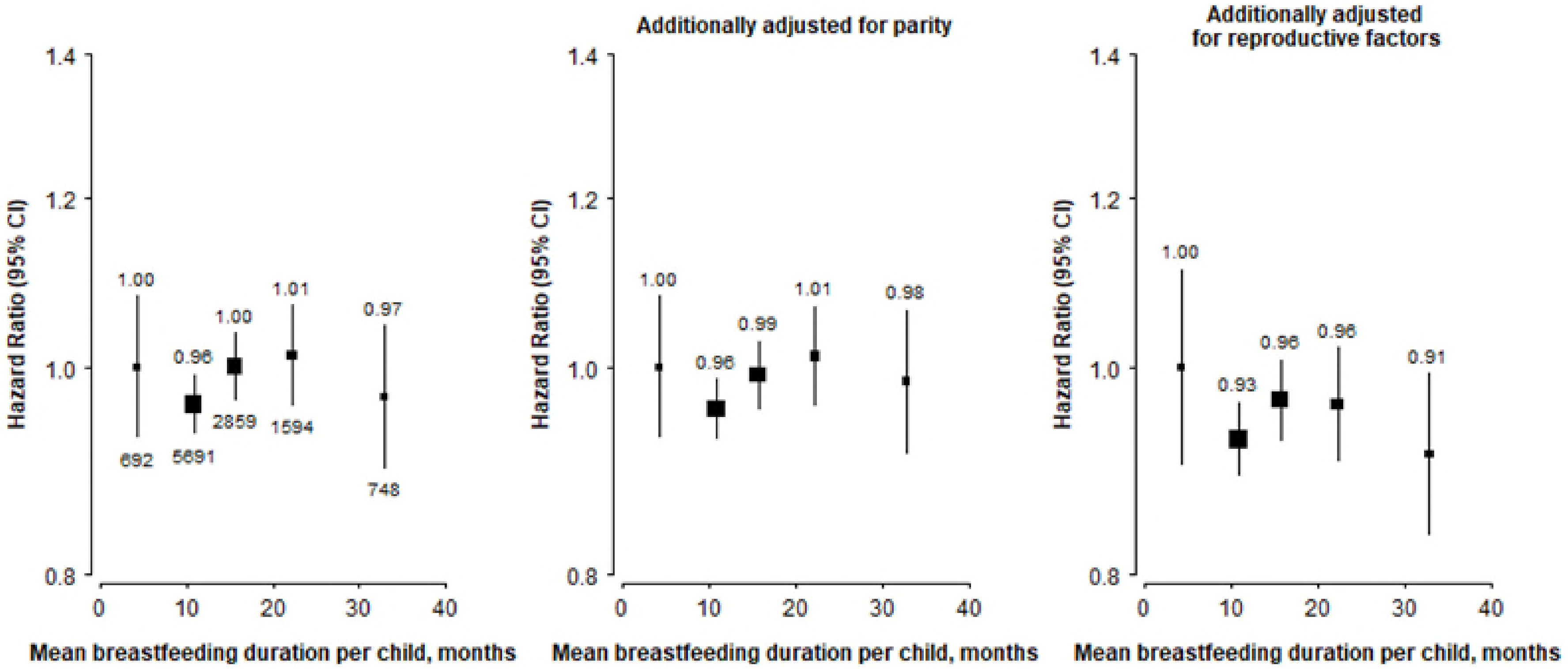

Supplemental Figure S7. Association of mean breastfeeding duration per child and lifetime breastfeeding duration with incident type 2 diabetes among ever breastfeeding parous women, by number of live births

Models were stratified by age-at-risk and study area and adjusted for education, household income, smoking status, alcohol drinking, physical activity, diet, and family history of diabetes. The area of each square is inversely proportional to the SE of the log risk. Vertical lines indicate corresponding 95% confidence intervals (CI). The numbers above the squares are the HRs and the numbers below the squares are the number of type 2 diabetes diagnoses in that group.

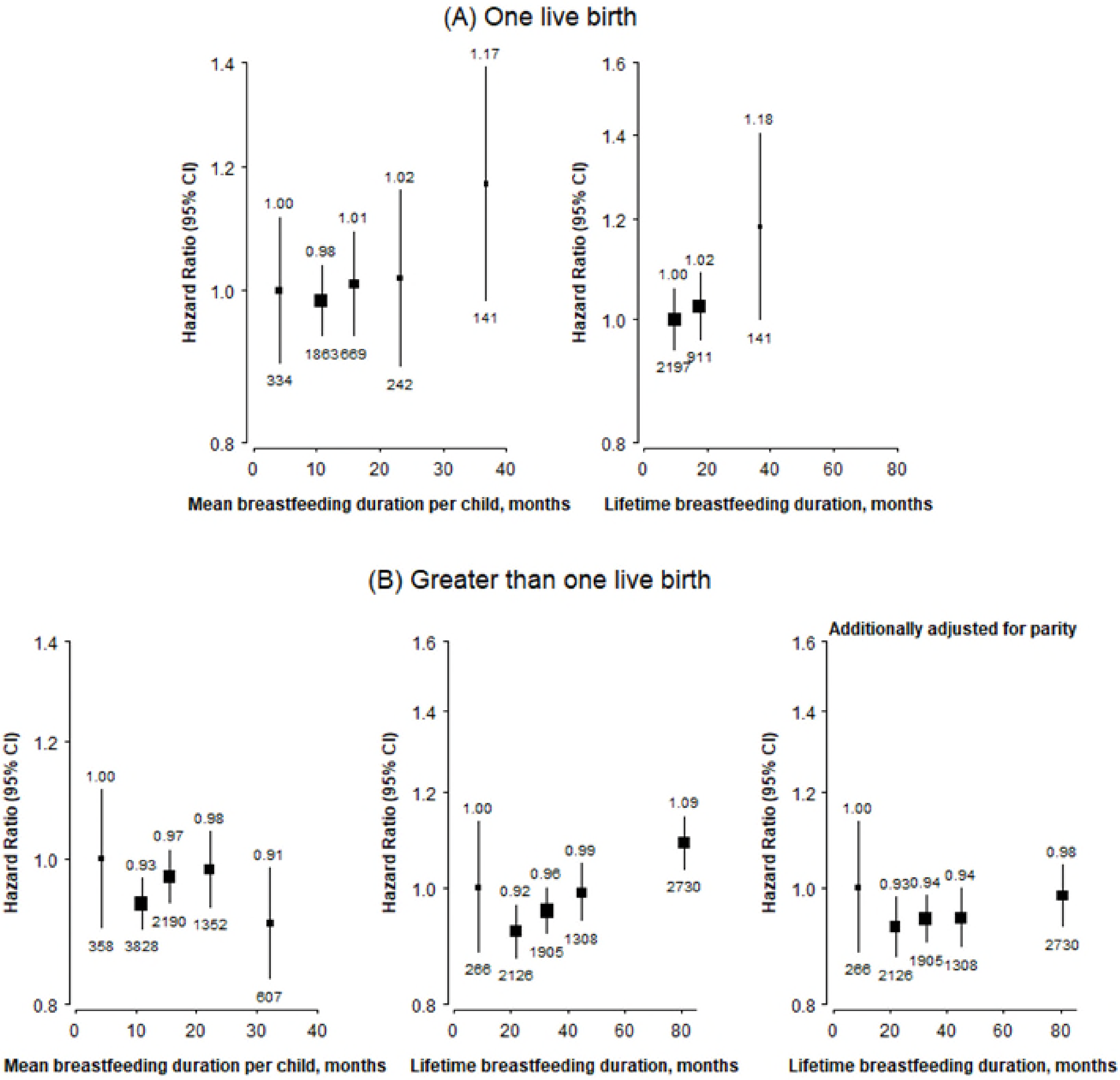

Supplemental Figure S8. Association of mean breastfeeding duration per child and lifetime breastfeeding duration with incident type 2 diabetes among ever breastfeeding parous women, by age at menarche

Models were stratified by age-at-risk and study area and adjusted for education, household income, smoking status, alcohol drinking, physical activity, diet, and family history of diabetes. The area of each square is inversely proportional to the SE of the log risk. Vertical lines indicate corresponding 95% confidence intervals (CI). The numbers above the squares are the HRs and the numbers below the squares are the number of type 2 diabetes diagnoses in that group.

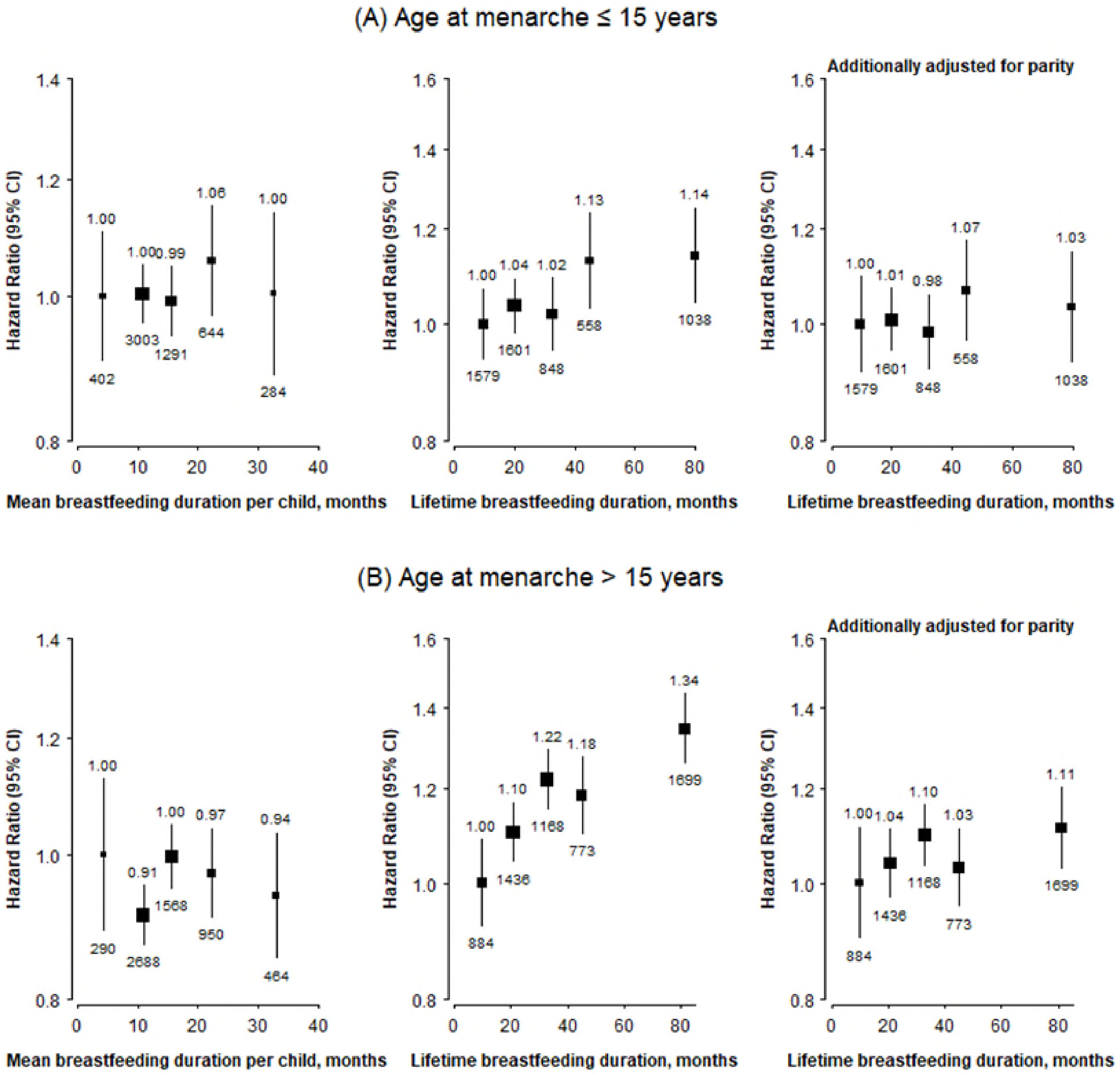

Supplemental Figure S9. Association of mean breastfeeding duration per child and lifetime breastfeeding duration with incident type 2 diabetes among ever breastfeeding parous women, by age at first birth

Models were stratified by age-at-risk and study area and adjusted for education, household income, smoking status, alcohol drinking, physical activity, diet, and family history of diabetes. The area of each square is inversely proportional to the SE of the log risk. Vertical lines indicate corresponding 95% confidence intervals (CI). The numbers above the squares are the HRs and the numbers below the squares are the number of type 2 diabetes diagnoses in that group.

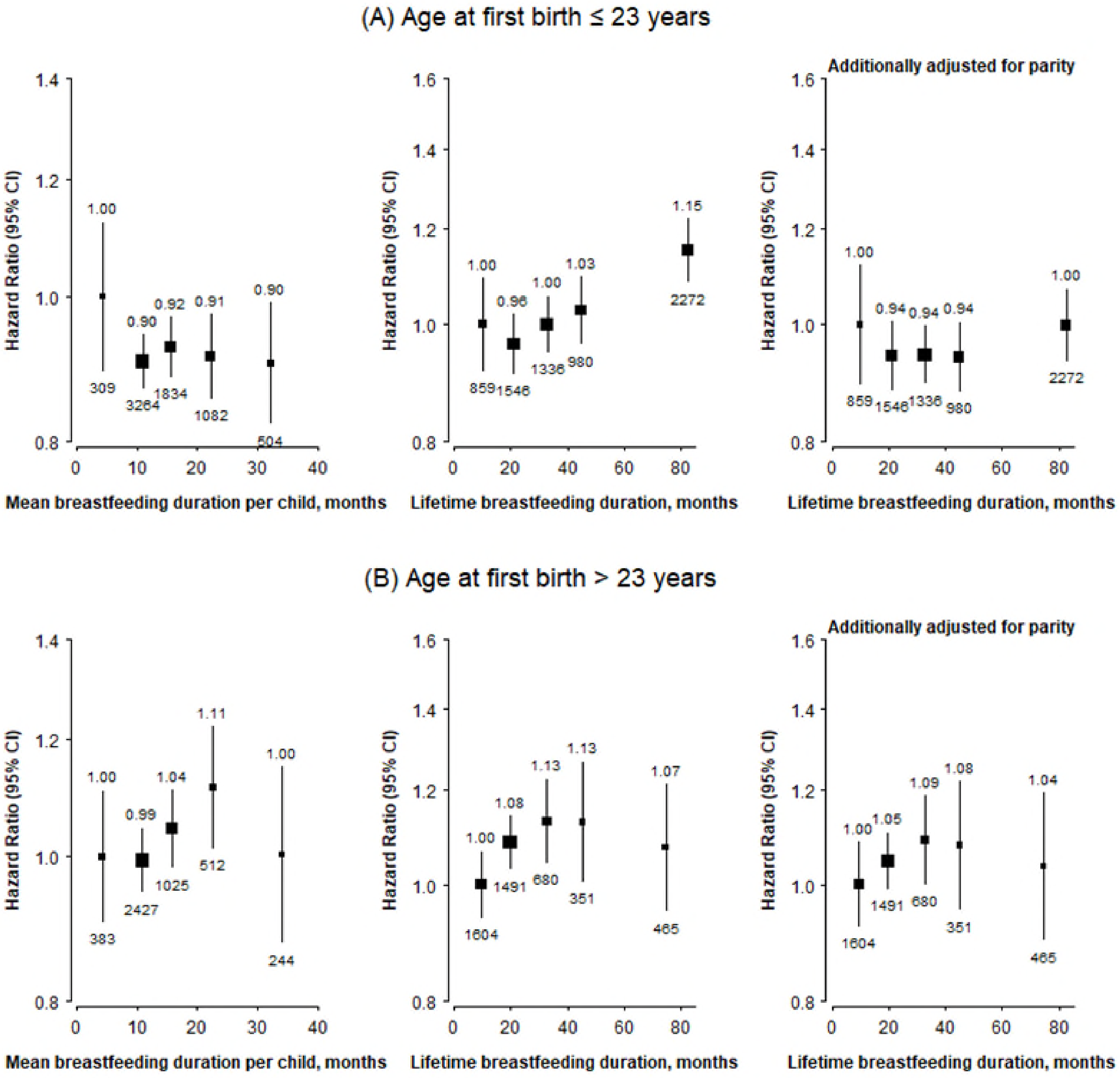

Supplemental Figure S10. Association of mean breastfeeding duration per child and lifetime breastfeeding duration with incident type 2 diabetes among ever breastfeeding parous women, by menopause status

Models were stratified by age-at-risk and study area and adjusted for education, household income, smoking status, alcohol drinking, physical activity, diet, and family history of diabetes. The area of each square is inversely proportional to the SE of the log risk. Vertical lines indicate corresponding 95% confidence intervals (CI). The numbers above the squares are the HRs and the numbers below the squares are the number of type 2 diabetes diagnoses in that group.

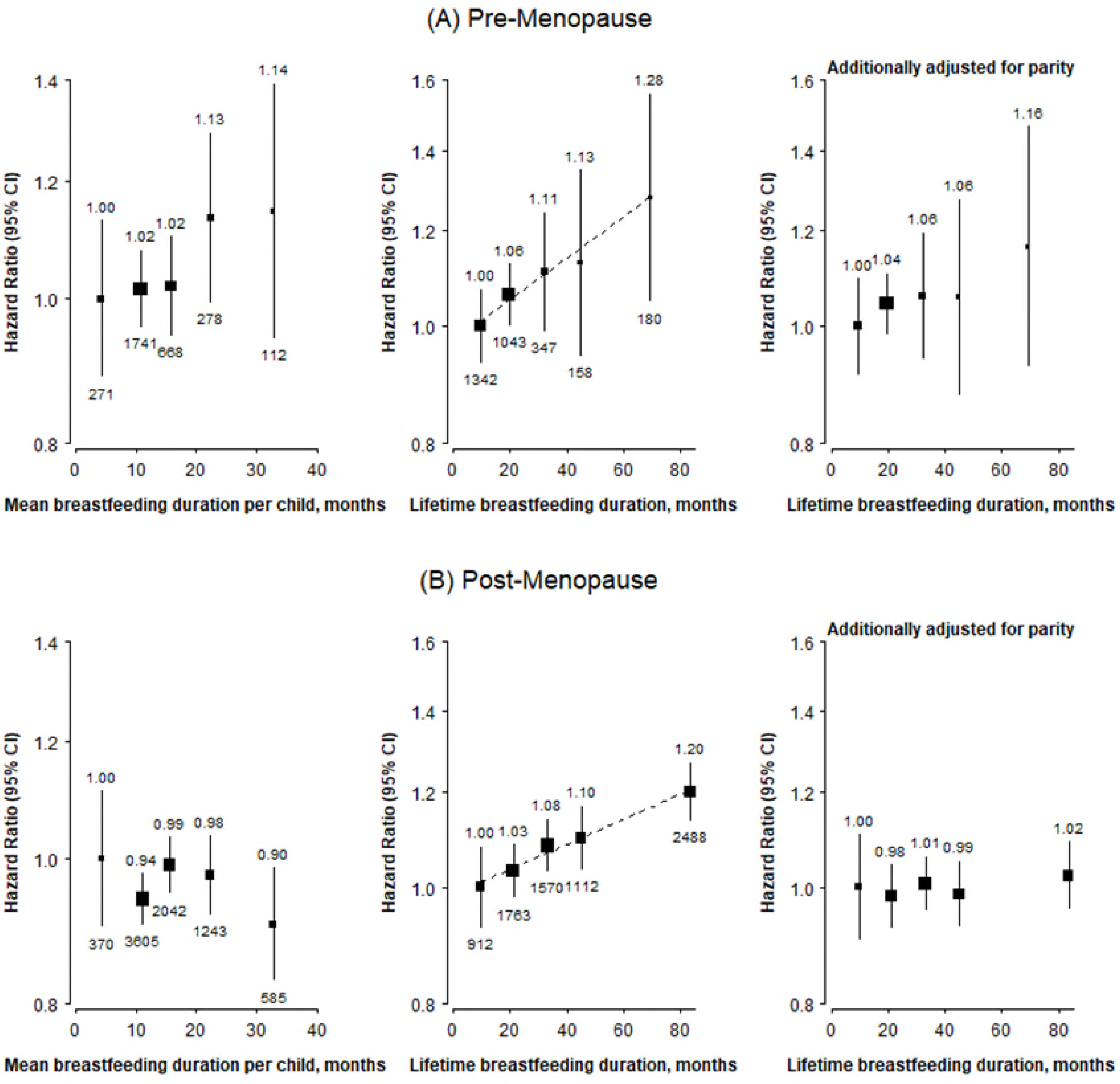

Supplemental Figure S11. Association of number of children with incident type 2 diabetes in men and women

Models were stratified by age-at-risk and study area and adjusted for education, household income, smoking status, alcohol use, physical activity, diet, and family history of diabetes. The area of each square is inversely proportional to the SE of the log risk. Vertical lines indicate corresponding 95% confidence intervals (CI). The numbers above the squares are the HRs and the numbers below the squares are the number of type 2 diabetes diagnoses in that group.

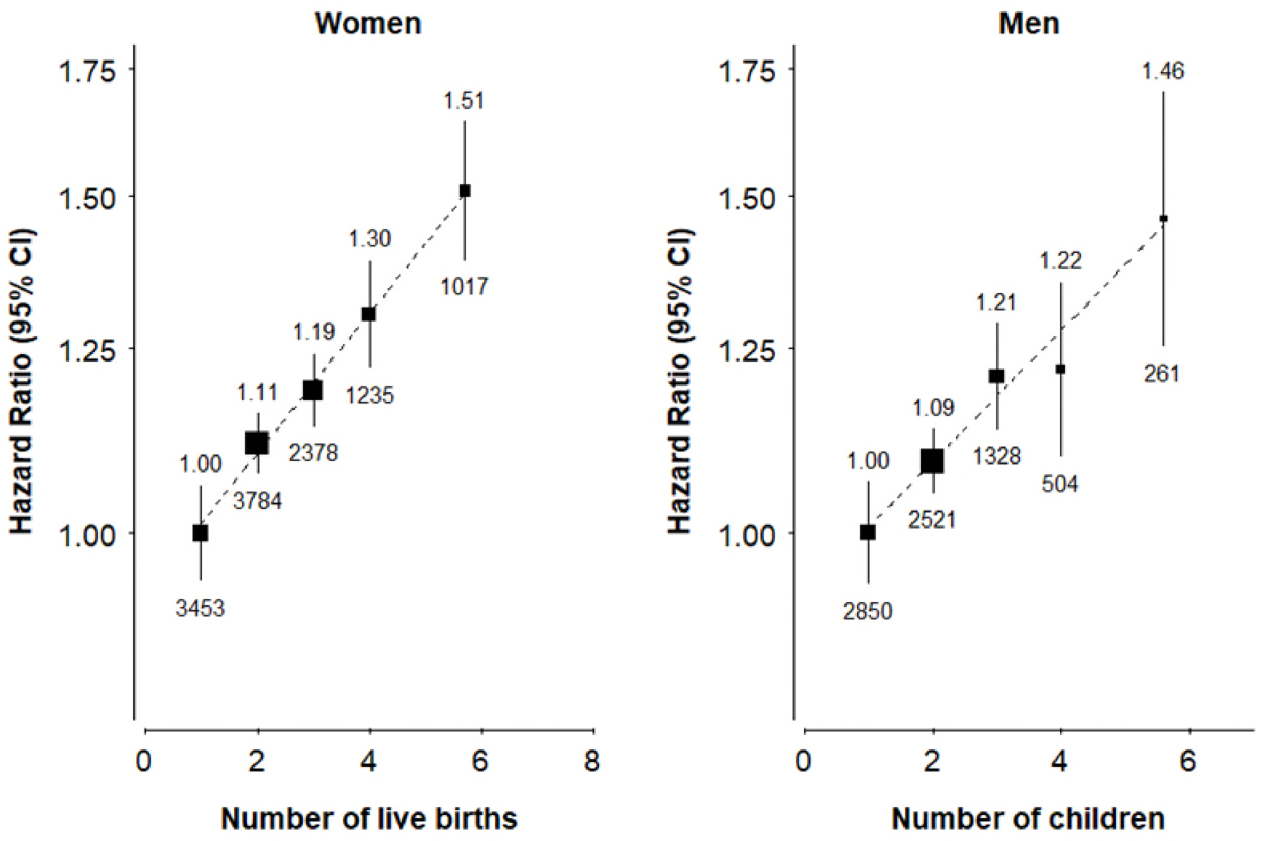

Supplement: online supplemental file 1 [file bmjopen-16-6-s001.pdf]
