# Supplementary material for: Breastfeeding and risk of maternal type 2 diabetes: a prospective cohort study of 280 000 women in China
Source: BMJ Open. 2026 Jun 28;16(6):e109377. doi: 10.1136/bmjopen-2025-109377 (PMC13311600; doi:10.1136/bmjopen-2025-109377)
Supplement: Supplementary file 2 [file bmjopen-2025-109377.reviewer_comments.pdf]

## PEER REVIEW HISTORY

BMJ Open publishes all reviews undertaken for accepted manuscripts. Reviewers are asked to complete a checklist review form and are provided with free text boxes to elaborate on their assessment. These free text comments are reproduced below.

### ARTICLE DETAILS

**Title (Provisional)**

Breastfeeding and risk of maternal type 2 diabetes: a prospective cohort study of 280,000 women in China

**Authors**

Kaminer, Johanna; Kartsonaki, Christiana; Sherliker, Paul; Yang, Ling; Iona, Andri; Pei, Pei; Chen, Yiping; Sun, Dianjianyi; Yang, Xiaoming; Lu, Yan; Du, Huaidong; Lv, Jun; Yu, Canqing; Chen, Junshi; Li, Liming; Chen, Zhengming; Bragg, Fiona; China Kadoorie Biobank Collaborative Group, on behalf of

---

### VERSION 1 - REVIEW

---

| | |
|---|---|
| **Reviewer** | **1** |
| **Name** | **Marschner, Simone** |
| **Affiliation** | **The University of Sydney, Westmead Applied Research Centre** |
| **Date** | **15-Feb-2026** |
| **COI** | **None** |

---

This paper is well written and the data has been analysed appropriately with sufficient detail.

I would suggest that the main aim be very clears. Are you more interested in comparing parous women with non-parous or breast feeding duration? I would be tempted to remove the non-parous women as it distracts.

The study population should be in the design section and not in the statistical analysis section and be clear. At the moment you only talk about the overall biobank rather than this studies subpopulation in that section.

At one point you examine under 6 months breast feeding which is important as this may be where the difference lies so you might want to do that throughout.

In the statistical methods section, please clarify if you are adjusting or stratifying as stratifying normally refers to different baseline hazards,

---

| | |
|---|---|
| **Reviewer** | **2** |
| **Name** | **Keenan-Devlin, Lauren** |
| **Affiliation** | **NorthShore University HealthSystem** |
| **Date** | **09-Mar-2026** |
| **COI** | **None** |

---

Thank you for the opportunity to review the manuscript "Breastfeeding and risk of maternal type 2 diabetes among 280,000 women in China." This secondary analysis of an existing database explores incidence of Type 2 diabetes among a large prospective cohort in association with breastfeeding. The manuscript adds to epidemiologic evidence of the association between breastfeeding and lifetime metabolic disease risk, particularly for the Chinese population where the authors highlight concerning increases in diabetes incidence in recent decades, as well as patterns of breastfeeding behavior that differ from other countries where this relationship has been more thoroughly studied.

The paper is overall well-written and well organized. The results appear to highlight that in a population that overwhelmingly breastfeeds, factors like SES and parity likely play a more important role in the development of DM in later life, as they showed an inverse relationship between lifetime breastfeeding years and DM that was totally attenuated by parity. I have some minor feedback, indicated below.

Introduction:

The introduction is clearly written and includes sufficient background for the reader to appreciate the contribution of this analysis to what is already known.

Methods:

The cohort used for this analysis is advantageous because of the completeness of data available on participants via linkage to numerous databases and diagnoses codes.

At the bottom of page 7 and top of page 8 the authors include categorical breastfeeding durations and other categorical variable options in parentheticals that are difficult to read as presented. First, it is unclear to me why breastfeeding was analyzed categorically unless duration was

reported categorically—this should be clarified. Next, I recommend editing all of these parentheticals for clarity, either by adjusting formatting (use of spaces?) or referencing a table where the options can be presented more clearly.

At the bottom of p8 the authors state that "Adjusted HRs…were compared across…" was this a statistical test, or were these examined in a stratified analysis? Please clarify.

Results:

I suggest using the term "participant" rather than "women" when reporting on subjects since this is a consented cohort and not a population or registry sample.

For a cohort born in the 50s and 60s the follow up time of 11 years is relatively short and could miss later onset of DM. This should be mentioned in the limitations section.

Conclusion:

At the bottom of p16 the authors note prior findings that parity is associated with DM risk in women and men and point to lifestyle factors—this requires a brief explanation. What factors associated with being a parent—for men as well as women—could account for elevated DM risk in later life? This seems to be one of the stand-out findings from this study.

---

## VERSION 1 - AUTHOR RESPONSE

***REVIEWER #1***

1. **I would suggest that the main aim be very clears. Are you more interested in comparing parous women with non-parous or breast feeding duration? I would be tempted to remove the non-parous women as it distracts.**

   Response – As noted in Reviewer 2's sixth comment, the reported associations of parity with risk of incident T2D are important findings. They provide important context for interpreting the presented associations of breastfeeding with incident T2D as well as their relevance for the burden of T2D among women in China. We therefore prefer to keep these analyses in the manuscript while retaining the clear focus on breastfeeding and breastfeeding duration as the main exposures, consistent with the stated aims of the study.

2. **The study population should be in the design section and not in the statistical analysis section and be clear. At the moment you only talk about the overall biobank rather than this studies subpopulation in that section.**

   Response – We have now renamed the "Study population" section as "China Kadoorie Biobank study design and population" to clarify that this is referring to the China Kadoorie

   Biobank study as a whole. Inclusion of a description of the full study population is important given reference to the association between the number of children and risk of incident T2D among men in the study. We consider derivation of the analysis population as part of the

statistical methods and therefore prefer to keep this in the “Statistical analyses” section.
However, we would be happy to revise the placement of this text within the manuscript if the Editor prefers.

3. **At one point you examine under 6 months breast feeding which is important as this may be where the difference lies so you might want to do that throughout.**

   Response – In the manuscript we assess risk of incident T2D when compared with a reference group comprising women with a mean breastfeeding duration per child of under 6 months and, where appropriate, we report risk of incident T2D per 6 months longer breastfeeding duration. A relatively small number of women in the study had a mean breastfeeding duration per child of under 6 months or a lifetime breastfeeding duration of under 6 months. We therefore did not include findings of analyses examining in detail the association of breastfeeding durations of less than 6 months with risk of incident T2D. As presented in Appendix 1 at the end of this response letter, and consistent with the overall conclusion of the manuscript, there was no apparent association with risk of maternal incident T2D at breastfeeding durations of 6 months or less. However, there is a high degree of uncertainty around the estimated incident T2D risks given the small number of women and of incident T2D diagnoses included in these analyses. We therefore prefer not to include these findings in the manuscript to avoid overwhelming the reader with further findings which provide little additional valuable insight.

4. **In the statistical methods section, please clarify if you are adjusting or stratifying as stratifying normally refers to different baseline hazards.**

   Response – Done. The current description of the Cox proportional hazards models, which were stratified by age-at-risk and study area and adjusted for other described variables including socioeconomic and lifestyle factors and family history of T2D, is correct. The use of stratified Cox regression models is a well-established statistical and epidemiological technique; as noted by the reviewer, this allows for a different baseline hazard in each stratum, such that the proportional hazards assumption was made only within strata of age group and study area. We have now included this additional clarification in the manuscript (page 8, lines 198-200).

***REVIEWER #2***

1. **At the bottom of page 7 and top of page 8 the authors include categorical breastfeeding durations and other categorical variable options in parentheticals that are difficult to read as presented. First, it is unclear to me why breastfeeding was analyzed categorically unless duration was reported categorically—this should be clarified.**

   Response – Done. We have now clarified that breastfeeding durations were examined as categorical variables in order to assess the shape of their associations with risk of incident T2D, where we now state, “In order to assess the shape of the association of breastfeeding duration with risk of incident T2D, lifetime breastfeeding duration (1-12/ 13-24/ 25-36/ 37-48/ >48 months) and breastfeeding duration per child (<7/ 7-12/ 13-18/ 19-24/ >24 months, respectively) were each classified into five groups” (page 8, lines 180-183).

2. **Next, I recommend editing all of these parentheticals for clarity, either by adjusting formatting (use of spaces?) or referencing a table where the options can be presented more clearly.**

   Response –Done (page 8, lines 180-183, 189-197, 200-202).

3. **At the bottom of p8 the authors state that "Adjusted HRs…were compared across…" was this a statistical test, or were these examined in a stratified analysis? Please clarify.**

   Response – Done. We have revised the wording to clarify that these associations were examined in stratified analyses, where we now state that "Adjusted HRs for incident T2D associated with breastfeeding duration were examined separately for female participants in urban and rural areas and in strata of birth cohort, BMI, and other reproductive factors, including number of live births, age at first birth and menopause status" (page 9, lines 209-212).

4. **I suggest using the term "participant" rather than "women" when reporting on subjects since this is a consented cohort and not a population or registry sample.**

   Response – Done. The term "women" has been replaced by "participants" or "female participants" wherever appropriate throughout the manuscript.

5. **For a cohort born in the 50s and 60s the follow up time of 11 years is relatively short and could miss later onset of DM. This should be mentioned in the limitations section.**

   Response – We agree that, among the cohort of women born in the 1950s and 1960s, the study follow-up may not fully capture later-onset T2D. However, women remaining in the study who were born during this period would be aged approximately 60-80 years at the global censoring date (January 2019). Although data specific to China on the average age at diagnosis of T2D are lacking, studies elsewhere have estimated average ages at diagnosis of between 50 and 65 years (PMID: 39587196, 33741554, 14532317). We would therefore expect a relatively small proportion of T2D diagnoses to be missed due to later onset, in particular given the tendency for a younger age at T2D diagnosis in the Chinese population.

   Moreover, Cox proportional hazards regression models were used in the present study, which appropriately account for differences in follow-up time and censoring. Nevertheless, we have now added reference to potential misclassification of T2D status in discussion of the study's limitations (please see our response to the Editors' fifth comment).

6. **At the bottom of p16 the authors note prior findings that parity is associated with DM risk in women and men and point to lifestyle factors—this requires a brief explanation. What factors associated with being a parent—for men as well as women—could account for elevated DM risk in later life? This seems to be one of the stand-out findings from this study.**

   Response – Done. We have now briefly elaborated on possible explanations for the association between number of children and risk of incident T2D among men and women, where we also reference a previous paper focused on this topic within the China Kadoorie Biobank study population should readers wish to explore this in more detail (page 18, lines 386-390).

**Appendix 1. Association among ever breastfeeding parous women of mean breastfeeding duration per child among women with a breastfeeding duration per child of 6 months or less, and of lifetime breastfeeding duration among women with a lifetime breastfeeding duration of 6 months or less**

Models were stratified by age-at-risk and study area and adjusted for education, household income, smoking status, alcohol drinking, physical activity, diet, and family history of diabetes. The area of each square is inversely proportional to the SE of the log risk. Vertical lines indicate corresponding 95% confidence intervals (CI). The numbers above the squares are the HRs.

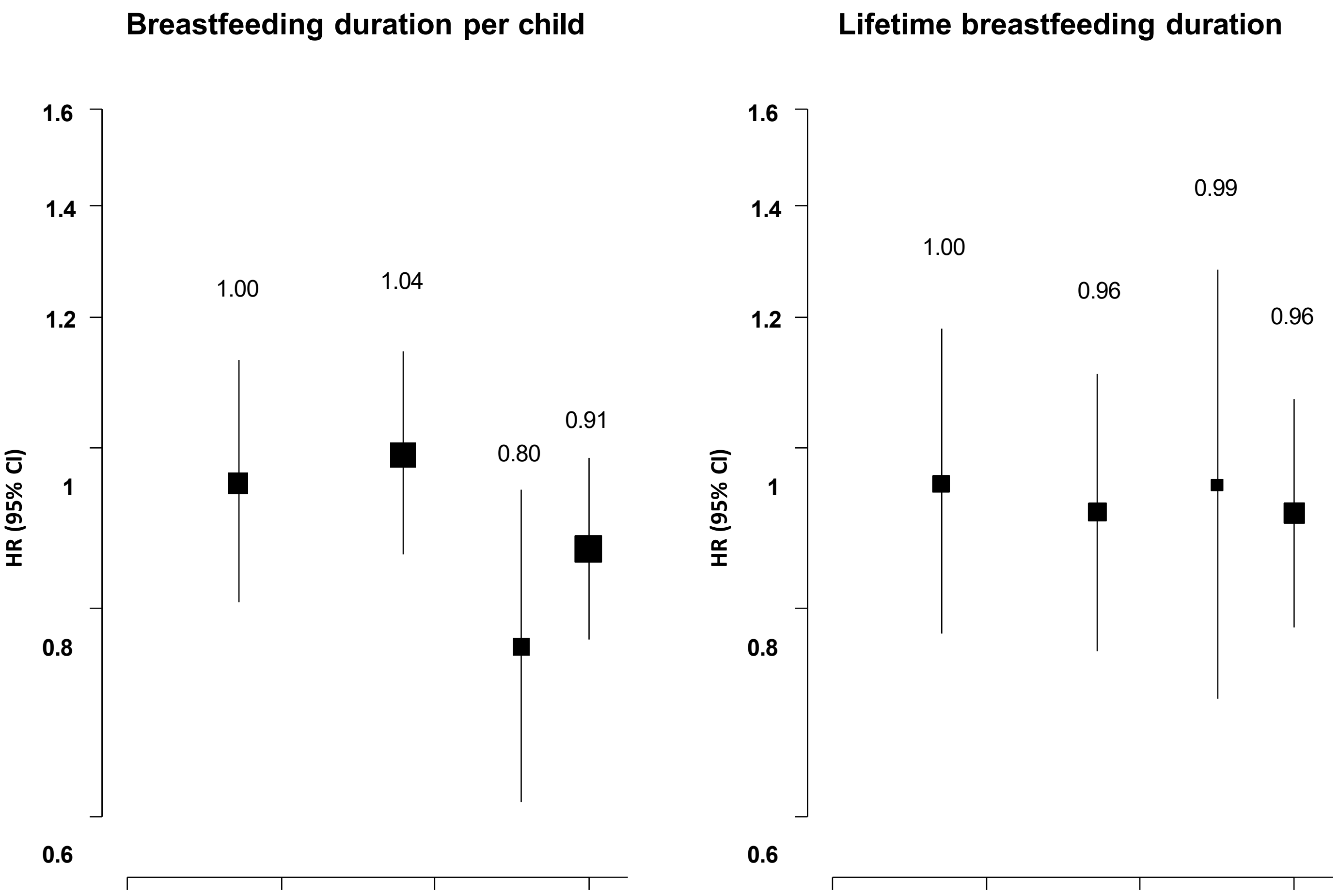

0 2 4 6

Mean breastfeeding duration per child, months

## VERSION 2 - REVIEW

| | |
|---|---|
| **Reviewer** | **1** |
| **Name** | **Marschner, Simone** |
| **Affiliation** | **The University of Sydney, Westmead Applied Research Centre** |
| **Date** | **20-May-2026** |
| **COI** | |

This paper is very thorough and very interesting.

My only concern is that most women were breastfeeding and for quite a long time. I think the conclusion that breast feeding has no association with T2D among Chinese women needs to be presented very carefully. Other studies in the West have many more women breastfeeding for much shorter durations and the protective effect may be evident when comparing under 6 months of lifetime breast feeding versus over 6 months rather than a linear effect that keeps increasing for this large number of months of breast feeding.

| | |
|---|---|
| **Reviewer** | **2** |
| **Name** | **Keenan-Devlin, Lauren** |
| **Affiliation** | **NorthShore University HealthSystem** |
| **Date** | **27-May-2026** |
| **COI** | |

5

All comments have been sufficiently addressed.

## VERSION 2 - AUTHOR RESPONSE

***<u>REVIEWER #1</u>***

1. **My only concern is that most women were breastfeeding and for quite a long time. I think the conclusion that breast feeding has no association with T2D among Chinese women needs to be presented very carefully. Other studies in the West have many more women breastfeeding for much shorter durations and the protective effect may be evident when comparing under 6 months of lifetime breast feeding versus over 6 months rather than a linear effect that keeps increasing for this large number of months of breast feeding.**

Response – We now describe the proportion of women reporting lifetime breastfeeding durations of less than 6 months in the present and previous study populations (lines 354-358) and acknowledge in the discussion of the study's limitations that, 'the small proportion of women who reported lifetime breastfeeding durations of less than 6 months prevented detailed investigation of the relevance of such durations for risk of T2D, and differing associations at these shorter durations cannot be excluded based on the presented findings' (lines 418-422). We have also revised the study conclusion to reflect this point; this now reads 'After controlling for key confounders, including parity, in this population in which most women were parous and reported breastfeeding for relatively long durations, we found no association of breastfeeding initiation or duration with incident T2D' (lines 429-432).

---
